# Conversion of Short and Medium Chain Fatty Acids into Novel Polyhydroxyalkanoates Copolymers by *Aeromonas* sp. AC_01

**DOI:** 10.3390/ma15134482

**Published:** 2022-06-25

**Authors:** Karolina Szacherska, Krzysztof Moraczewski, Sylwester Czaplicki, Piotr Oleskowicz-Popiel, Justyna Mozejko-Ciesielska

**Affiliations:** 1 Department of Microbiology and Mycology, Faculty of Biology and Biotechnology, University of Warmia and Mazury in Olsztyn, 10-719 Olsztyn, Poland; karolina.szacherska@uwm.edu.pl; 2 Institute of Materials Engineering, Kazimierz Wielki University, 85-064 Bydgoszcz, Poland; kmm@ukw.edu.pl; 3 Department of Plant Food Chemistry and Processing, Faculty of Food Sciences, University of Warmia and Mazury in Olsztyn, Pl. Cieszyński 1, 10-726 Olsztyn, Poland; sylwester.czaplicki@uwm.edu.pl; 4 Water Supply and Bioeconomy Division, Faculty of Environmental Engineering and Energy, Poznan University of Technology, 60-965 Poznan, Poland; piotr.oleskowicz-popiel@put.poznan.pl

**Keywords:** *Aeromonas* sp., biopolymers, medium chain fatty acids, PHAs, PHA copolymers, polyhydroxyalkanoates, short chain fatty acids

## Abstract

Polyhydroxyalkanoates (PHAs) production by *Aeromonas* sp. AC_01 was investigated using synthetic and waste derived short and medium chain fatty acids (SMCFAs). The obtained results revealed that the analyzed bacterial strain was able to grow and synthesize PHAs using SMCFAs. The highest PHA productivity was observed in the cultivation supplemented with a mixture of acetic acid and butyric acid (3.89 mg/L·h). Furthermore, SMCFAs-rich stream, derived from acidogenic mixed culture fermentation of acid whey, was found to be less beneficial for PHA productivity than its synthetic mixture, however the PHA production was favored by the nitrogen-limited condition. Importantly, *Aeromonas* sp. AC_01 was capable of synthesizing novel scl-mcl copolymers of 3-hydroxybutyrate (3HB), 3-hydroxyvalerate (3HV), 3-hydroxytridecanoate (3HtriD) and/or 3-hydroxytetradecaonate (3HTD) with high 3HB and 3HV fractions. They were identified with alterable monomers composition depending on the culture conditions used. Moreover, in-depth thermal analyses proved that they are highly resistant to thermal degradation regardless of their monomeric composition. The obtained results confirm that *Aeromonas* sp. AC_01 is a promising candidate for the biotechnological production of PHAs from SMCFAs with thermal properties that can be tuned together with their chemical composition by the corresponding adjustment of the cultivation process.

## 1. Introduction

In the last few decades, plastics have become an integral part in our daily life. Due to their persistence and accumulation in the ecosystems as a harmful waste, it is crucial to replace petrochemical-derived compounds with biodegradable, biobased and environmentally friendly polymers derived from renewable resources. Among biopolymers, polyhydroxyalkanoates (PHAs) are particularly a promising alternative to conventional plastics. They are produced by different microorganisms from a range of substrates under nutrient-limited conditions such as nitrogen, phosphorus or oxygen [1]. They are classified into short-chain-length PHA (scl-PHA) containing 3–5 carbon atoms per monomer, medium-chain-length PHA (mcl-PHA) with 6–14 carbon atoms per monomer, and long-chain-length (lcl-PHA) which contains more than 15 carbon atoms [2]. The best studied molecules are poly(3-hydroxybutyrate) homopolymer [P(3HB)] and poly(3-hydroxybutyrate-co-3-hydroxyvalerate) copolymer [P(3HB-co-3HV)] [3]. The potential usage of P(3HB) is limited due to its high crystallinity, stiffness, and brittleness. The incorporation of 3-hydroxyvalerate (3HV) monomer into P(3HB) improves its thermomechanical properties making this material more robust and flexible [4]. Furthermore, copolymers are known to biodegrade faster than homopolymers due to the presence of the inherited amorphous region. It was proved that as the 3HV content in the copolymer increases, the crystallinity decreases and the biodegradation rate improves [5]. One of the key challenges in microbial PHA production is to make this biotechnological process economically feasible. High production costs could be reduced by using waste-derived streams as organic substrates and novel microbial strains able to metabolize such carbon sources into the valuable biopolymers. Transforming waste into valuable products is a revolutionary waste management path and an environmentally friendly process [6]. In recent years, a continuous increase in organic and biomass waste has been observed. It was estimated that sewage sludge from wastewater treatment contains around 50–80% of organic, toxic, and perishable contaminants that in high quantity poses a risk to the ecosystem [7,8]. Furthermore, acid whey constitutes also a serious environmental problem and is estimated that its global production reaches 180–190 million tons. Moreover, its worldwide volume is expected to rise in the coming years [9]. Sweet whey, a by-product from hard cheese production, is turned into valuable products such as protein powder. Acid whey, on the other hand, a by-product from the manufacture of Greek yogurt, cottage cheese, and the like, constitutes a serious environmental problem and its disposal is associated with a fee [10]. Therefore, SMCFAs that are produced from sludge and acid whey, and could be a feedstock for biopolymers synthesis by microorganisms make the bioprocess of PHA production more sustainable and eco-friendlier. It was previously shown that waste organic compounds from a wide range of waste streams can be used to produce short and medium chain fatty acids (SMCFAs) by acidogenic anaerobic mixed culture fermentation (MCF) as a platform towards PHA production [11,12]. So far, the synthetic SMCFAs have been successfully used as carbon sources in the cultivations of *Pseudomonas oleovorans* [13]; *Pseudomonas* sp. ST2 [14]; *Ralstonia eutropha* [15,16] and *Haloferax mediterranei* [17]. However, there are only a few reports describing the ability of pure bacteria cultures to convert waste-derived SMCFAs into PHAs [6,18,19,20].

Bacteria belonging to *Aeromonas* genera are Gram-negative, heterotrophic rods that can produce PHAs even under toxic environmental conditions [21]. Due to their robust growth and simple nutritional requirements, they are attractive targets for potential PHA production using different waste. *Aeromonas* sp. AC_01 was originally isolated from waste activated sludge in Poland. We previously published a brief report indicating that *Aeromonas* sp. AC_01 was capable of synthesizing PHAs using crude glycerol from biodiesel production [22]. Up to date, no effort has been made to investigate the ability of any *Aeromonas* strains to produce PHAs from waste-derived SMCFAs. However, *Aeromonas* sp. have been used for PHA synthesis using other substrates [23]. Therefore, the aim of this study was to explore a new, sustainable production of PHAs using SMCFAs by this highly interesting bacterium. *Aeromonas* sp. AC_01 was assayed to evaluate their capability to biosynthesize PHA while growing on SMCFAs from whey wastewater biodigestion effluent as well as single synthetic pure acids and their synthetic mixtures as carbon sources in the growth media.

## 2. Materials and Methods

### 2.1. Microorganism

*Aeromonas* sp. AC_01 was obtained from the Culture Collection of the Department of Microbiology and Mycology (University of Warmia and Mazury in Olsztyn, Olsztyn, Poland). It was isolated from waste activated sludge collected in spring from the aeration tank at the municipal sewage treatment plant “Łyna” in Olsztyn (Poland). The genetic analysis based on the 16S *rRNA* gene sequence revealed that this isolate exhibited the highest similarity with *Aeromonas hydrophila* [22].

### 2.2. Culture Conditions for PHA Biosynthesis

Before inoculation into the fermentation shake flasks, cells of *Aeromonas* sp. AC_01, maintained as a cryo-conserved culture at −80 °C in a glycerol stock, were grown in lysogeny broth (10 g/L tryptone, 5 g/L yeast extract, 10 g/L NaCl, pH 7.0) at 30 °C for 16 h with 150 rpm shaking. For PHA production, 250-mL Erlenmeyer flasks were inoculated with 5% (*v*/*v*) of the bacterial seed. The analyzed strain was cultivated in two different mineral salt media (MSM): non-limited MSM (7 g/L KH_2_PO_4_, 3.5 g/L Na_2_HPO_4_·12H_2_O, 10 g/L (NH_4_)_2_SO_4_) and nitrogen-limited MSM (N-limited MSM) (7 g/L KH_2_PO_4_, 3.5 g/L Na_2_HPO_4_·12H_2_O, 1 g/L (NH_4_)_2_SO_4_). Each medium was supplemented with MgSO_4_·7H_2_O (1 g/L) and trace element solution (2.5 mL/L) consisting of 20 g/L FeCl_3_·6H_2_O, 10 g/L CaCl_2_·H_2_O, 0.03 g/L CuSO_4_·5H_2_O, 0.05 g/L MnCl_2_·4H_2_O, 0.1 g/L ZnSO_4_·7H_2_O dissolved in 0.5 N HCl. The experiments were conducted using (as the only carbon source): (A) various concentrations (1, 2 and 3 g/L) of single synthetic SMCFAs: acetic (HAc), butyric (HBu), valeric (HVa) and caproic acid (HCa); (B) different ratios of HAc and HBu (g/L): 1.6:0.4; 1.2:0.8; 0.8:1.2; 0.4:1.6 (C) various concentrations (10, 20 and 30% *v*/*v*) of a mixture of synthetic SMCFAs (SMCFAs_synthetic_-rich stream) to simulate a real SMCFAs-rich stream: acetic acid (2.85 g/L), butyric acid (9.86 g/L), valeric acid (0.16 g/L), caproic acid (3.05 g/L; (D) various concentrations (10, 20 and 30% *v*/*v*) of a mixture of SMCFAs received (SMCFAs_extracted_-rich stream) from the acidogenic anaerobic mixed culture fermentation of acid whey obtained from a crude cheese production line. The SMCFAs production was conducted in an up-flow anaerobic sludge blanket (UASB) bioreactor constructed from cylindrical plexiglass with recirculation ensuring biomass suspension as described by Duber et al. [9]. The effluent for PHAs production was taken from a steady-state phase with a dominant butyrate production. The sample of the effluent was filtered and the supernatant was analyzed by high-performance liquid chromatography (HPLC) to evaluate its composition (Table 1).

The pH of the growth media was adjusted to 7.0 by addition of 1M NaOH and 1M HCl. All cultivations were incubated at 30 °C and 150 rpm using rotary shaker. The cultivations were conducted for 48 h (with SMCFAs_synthetic_-rich stream and SMCFAs_extracted_-rich stream) or for 72 h (with single synthetic SMCFAs and different ratios of HAc and HBu). The samples for analyses were harvested at 24 h, 48 h and 72 h (only from cultivations with single synthetic SMCFAs and different ratios of HAc and HBu). All cultivations were conducted in triplicate.

### 2.3. Bacterial Growth and PHA Quantification

Cell growth was controlled by estimating cell dry mass (CDM). The culture samples were centrifuged at 9000× *g* for 10 min. The supernatant was removed and the biomass pellet was lyophilized for 24 h using Lyovac GT2 System (SRK Systemtechnik GmbH, Riedstadt, Germany). PHAs were extracted by shaking lyophilized cells in hot chloroform (50 °C) for 3 h. Next, the prepared mixture was filtered through No. 1 Whatman filter paper to remove cell debris. After filtration, the biopolyesters dissolved in chloroform were precipitated with 70% cold methanol and then allowed to evaporate at room temperature. The precipitated PHAs were quantified directly by the gravimetric method. The PHAs content was defined as the percentage of the ratio of PHAs concentration to total bacterial cells concentration.

### 2.4. Determination of Monomeric Composition by Gas Chromatography/Mass Spectrometry Analysis

To evaluate the PHAs composition, the lyophilized cells were analyzed by gas chromatography coupled with mass spectrometry (GC-MS QP2010 PLUS, Shimadzu, Kyoto, Japan) according to the method described previously by Możejko-Ciesielska and Pokój [22]. Shortly, the freeze-dried bacterial cells were suspended in a chloroform-methanol-sulfuric acid mixture (100/97/3, *v*/*v*/*v*). The methanolysis was conducted by heating the vials at 100 °C for 20 h. Then, the sulfuric acid was neutralized with Na_2_CO_3_ and the resulting mixture was dried with anhydrous Na_2_SO_4_. After filtration, the methyl esters were analyzed using a BPX70 (25 m × 0.22 mm × 0.25 mm) capillary column (SGE Analytical Science, Victoria, Australia) with helium as a carrier gas at a flow rate of 1.38 mL/min. The column was heated from 80 to 240 °C at a rate of 10 °C/min. The interface and ion source temperatures of GC-MS was set at 240 °C with the electron energy of 70 eV within the 45–500 *m/z* range. Identification of the mass signals were analyzed by comparison with pure 3-hydroxyacids standards (Larodan, Solna, Sweden): 3-hydroxyhexanoate (3HHx), -octanoate (3HO), -nonanoate (3HN), -decanoate (3HD), -undecanoate (3HuD), -dodecanoate (3HDD), -tridecanoate (3HtriD), tetradecanoate (3HTD).

### 2.5. PHAs Spectroscopic Studies

The PHAs spectroscopic studies were performed using a Fourier Transform Spectrophotometer (FTIR) Nicolet iS10 (ThermoScientific, Waltham, MA, USA) by attenuated total reflection method (ATR-FTIR) on samples without prior preparation. FTIR spectra were recorded in the range from 4000 to 650 cm^−1^. Each spectrum analyzed was the average of 16 recorded measurements.

### 2.6. Analysis of PHAs Thermal Properties

The thermal properties of the extracted PHAs were determined using differential scanning calorimetry (DSC) and thermogravimetry (TG). DSC tests were conducted using a Q200 differential scanning calorimeter (TA Instruments, New Castle, DE, USA) in a nitrogen atmosphere, temperature range from −70 to 230 °C and heating/cooling rate of 10 °C/min. Samples of approximately 1 mg were first rapidly heated to 230 °C and conditioned for 1 minute to remove the thermal history of the material. Pre-heating was also aimed at homogenizing the sample, which due to its characteristics consisted of several layers of the same material. The samples were then cooled to −70 °C and re-heated to 230 °C. Thermal analysis of the PHAs was based on the second heating curve, from which the glass transition temperature (T_g_), cold crystallization temperature (T_cc_), change in enthalpy of cold crystallization (ΔH_cc_), melting point (T_m_) and change in enthalpy of melting process (ΔH_m_) were evaluated.

TG tests were performed using a Q500 thermobalance (TA Instruments, New Castle, DE, USA) in a nitrogen atmosphere, temperature range from −70 to 230 °C and heating rate of 10 °C/min. The mass of the analyzed samples was approximately 1 mg. From the thermogravimetric curve 5% mass loss temperature (T_5%_) was determined. The value of T_5%_ was used as a parameter determining the start of thermal degradation of the material, taken as the thermal resistance of the biomaterial. The TG analysis was also completed with the determination of the mass loss onset temperature (T_ons_) of the main stage of degradation of the tested PHAs.

### 2.7. Statistical Analysis

STATISTICA v.13.1 (StatSoft, Inc., Tulsa, OK, USA) was used to conduct statistical analysis of all data. All samples were analyzed in triplicate. The Mann–Whitney U-test was used to determine the significance of differences in PHAs concentration between experimental variants. Data were reported as the median ± SD at the significance level of *p* < 0.05.

## 3. Results and Discussion

### 3.1. Biomass Concentration and PHAs Production Using Individual Synthetic SMCFAs

In the first stage of the study, typical SMCFAs products of anaerobic acidification (acetic acid, butyric acid, valeric acid, and caproic acid) were used separately to investigate the ability of *Aeromonas* sp. AC_01 strain to metabolize them into PHAs. They were tested at the initial concentrations of 1.0, 2.0, and 3.0 g/L. High initial carbon source concentrations were speculated to be beneficial to achieve high cell densities and PHAs accumulation [24]. However, SMCFAs could be toxic for bacterial cells. At higher concentrations (over 3.0 g/L) these synthetic SMCFAs were acting as inhibitors for *Aeromonas* sp. AC_01 growth (data not shown). Also, Zhang et al. [25] reported a strong inhibition of *Aeromonas hydrophila* 4AK4 growth when the culture medium was supplemented with valerate at the concentration over 3 g/L.

**Figure 1:**
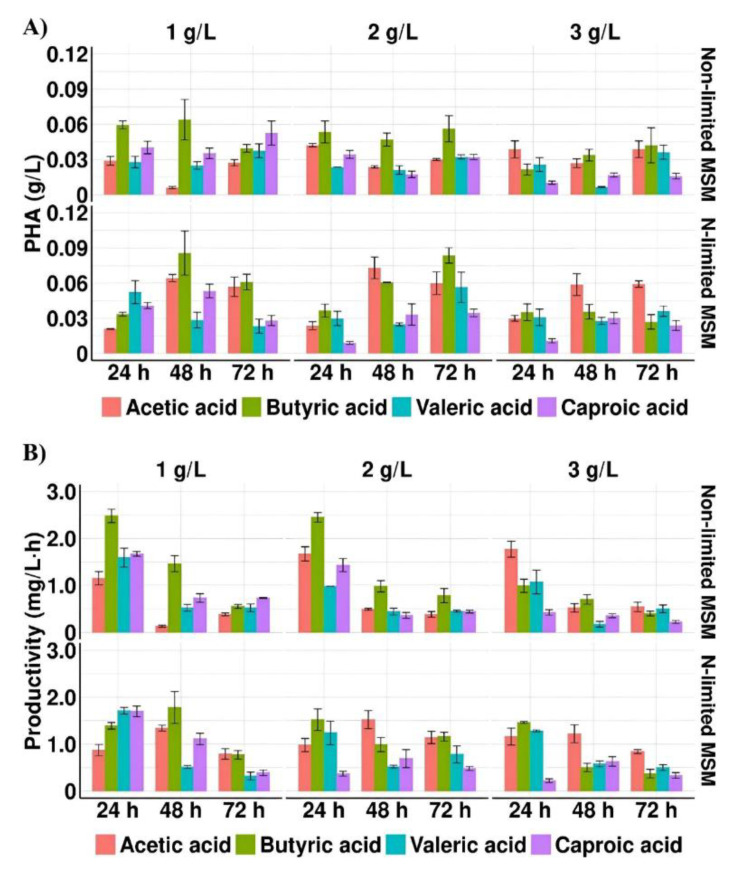
(**A**) PHA concentration and (**B**) PHA productivity profiles of *Aeromonas* sp. AC_01 cultured in the medium supplemented with individual short and medium chain fatty acids at the concentration of 1 g/L, 2 g/L and 3 g/L Abbreviations: non-limited MSM, non-limited mineral salt medium; N-limited MSM, nitrogen limited mineral salt medium.

As it is shown in Figure 1A,B and Table 2, all tested SMCFAs in all concentrations supported the bacterial growth and PHA production. The results indicated that SMCFAs concentration considerably impacted the growth of bacteria. Only the increase in acetic acid concentration caused an increase in the biomass production, whereas in the cultures with more than 1 g/L of butyric acid, valeric acid or caproic acid a decreased cell concentration was revealed.

The results showed that only in the cultures with acetic acid pH of the medium in all measured time-points was alkaline (pH > 7) (Table 2). In these cultivations where the highest biomass concentration was observed, it could suggest that the high consumption of acetate caused a pH increase [26]. Whereas in the cultivations with other acids the CDM values were lower due to their inhibitory effect, thus bacteria were not able to consume them effectively. Moreover, it was assumed that an alkaline growth medium could positively affect the tolerance level of *Aeromonas* sp. AC_01 to high acetic acid concentration. Whereas the pH values of the media with other tested SMCFAs were below 7 in all measured time-points, therefore the severe effect of the high concentrations of butyric, valeric and caproic acids may not be eliminated. The same observation was made by Gao et al. [27] who proved that an alkaline environment could effectively alleviate growth inhibition of *Yarrowia lipolytica* during its cultivations with SMCFAs towards lipid biosynthesis. The highest biomass concentration (2.0 g/L) was evaluated in the culture with 3.0 g/L of acetic acid under non-limited condition at 72 h. Lesser value (about 1.6 g/L) was detected when *Aeromonas* sp. AC_01 was grown on 1.0 g/L of butyric acid under the same condition. The results indicated that in all cultivations supplemented with valeric and caproic acid, regardless of the culture condition used, the biomass concentration was below 1.0 g/L.

The results suggested that nitrogen limitation affected positively PHA concentration. The highest PHA concentration was obtained with 1 g/L of butyric acid and 2 g/L of acetic acid under N-limited conditions (MSM with 1 g/L (NH_4_)_2_SO_4_)), 0.09 and 0.07 g/L, respectively. The statistically significant difference (*p* = 0.017) was reported between cultures with acetic and butyric acid under non-limited conditions (MSM with 10 g/L (NH_4_)_2_SO_4_). The reported observations were similar to those achieved by Anburajan et al. [21] who reached higher PHA yield cultivated *Aeromonas hydrophila* ATCC7966 in the famine phase compared to feast phase. Moreover, our data revealed that when valeric acid or caproic acid were used as the sole carbon source, PHAs were synthesized at lower concentrations (below 0.06 g/L) compared with acetic and butyric acid (*p* = 0.042 and *p* = 0.022, respectively). Lower PHA yield (0.03 g/L) was also achieved by Shi et al. [28] who cultured engineered *Aeromonas hydrophila* 4AK4 harboring overexpressed genes encoding β-ketothiolase and acetoacetyl-CoA reductase grown with yeast extract and acetate. However, further metabolic engineering through overexpression of *pta-ackA* gene was proved to strengthen the utilization of acetic acid and consequently PHA yield increased to 0.57 g/L.

The highest PHA productivity was observed in the cultures under non-limited conditions at 24 h when the growth medium was also supplemented with butyric acid (2.48 mg/L·h) and acetic acid (1.77 mg/L·h). The PHA productivity decreased about 23% and 30% at 72 h compared to 24 h in the cultures with butyrate and acetate, respectively. Also, *Ralstonia eutropha* ATCC 17699 showed the capability of producing PHAs on acetic, propionic and butyric acid, however the biopolymer productivity reached only 0.013, 0.036, and 0.037 mg/L·h, respectively [15]. The addition of glucose in the cultivation of this bacterial strain with propionic acid enhanced the PHA productivity to 0.74 mg/L·h [29].

### 3.2. Cell Dry Weight Concentration and PHA Production Using a Mixture of Acetic and Butyric Acid

**Figure 2:**
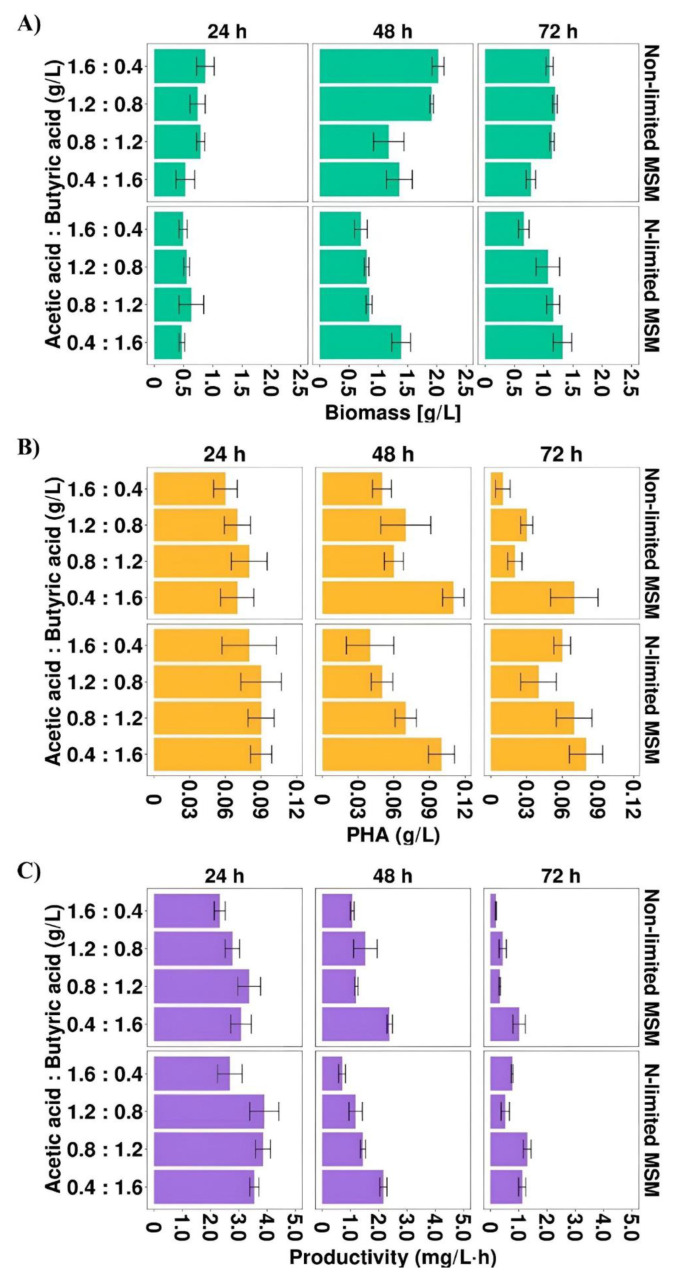
(**A**) Biomass concentration, (**B**) PHA concentration and (**C**) PHA productivity profiles of *Aeromonas* sp. AC_01 cultivated on the mixture of acetic and butyric acid. Abbreviations: non-limited MSM, non-limited mineral salt medium; N-limited MSM, nitrogen limited mineral salt medium.

As it was described above, acetic and butyric acids were the most preferable carbon sources that effectively supported *Aeromonas* sp. AC_01 growth, PHA concentration and productivity. Therefore, in the second stage of the study, the combinations of both acids were prepared to determine their effects as carbon sources on the biomass production and PHA biosynthesis level. Biomass concentration, PHA concentration and productivity were improved (Figure 2A–C) compared to the results obtained in the cultures with single acetic or butyric acid.

The results confirmed that the highest biomass concentration (2.02 g/L) was observed in the cultivation under non-limited conditions at 48 h with the highest proportion of acetic acid (1.6 HAc:0.4 HBu). This value was higher compared to those received from the cultures at the same time point with single acetic acid and butyric acid, the maximum biomass concentration reached 1.9 and 0.7 g/L, respectively. In addition, the results indicated that the mixture of both acids supported better PHA production than individual acids. The PHA concentration and productivity depended on the ratio of these SMCFAs (Figure 2B,C). Nevertheless, the highest PHA concentration was observed at 48 h in the cultivations under non-limited and N-limited conditions with the highest proportion of butyric acid (0.11 and 0.10 g/L, respectively). These values were also higher than those obtained in the cultures with single acids. As can be observed in Figure 2C, nitrogen limitation positively influenced on PHA productivity. In the cultivations with the mixture in the proportion of 1.2 HAc:0.8 HBu the PHAs were produced in the highest efficiency, reaching 3.9 mg/L·h at 24 h. At 48 and 72 h, in all experimental variants, PHA productivity decreased. Higher PHA productivity was reported by Gonzalez and Winterburn [30] who observed that when in the fermentation of *Haloferax mediterranei* the mixture of butyric and pentanoic acid was used at the constant concentration of 0.25 M the process productivity was improved and reached up to 12.8 mg/L·h. The same bacterial species produced PHA with lower productivity (0.010 mg/L·h) when was cultured on the mixture of butyric acid, valeric acid and Tween 20 [17].

### 3.3. Growth and PHA Production Using a SMCFAs_extracted_-Rich Stream and SMCFAs_synthetic_-Rich Stream

**Figure 3:**
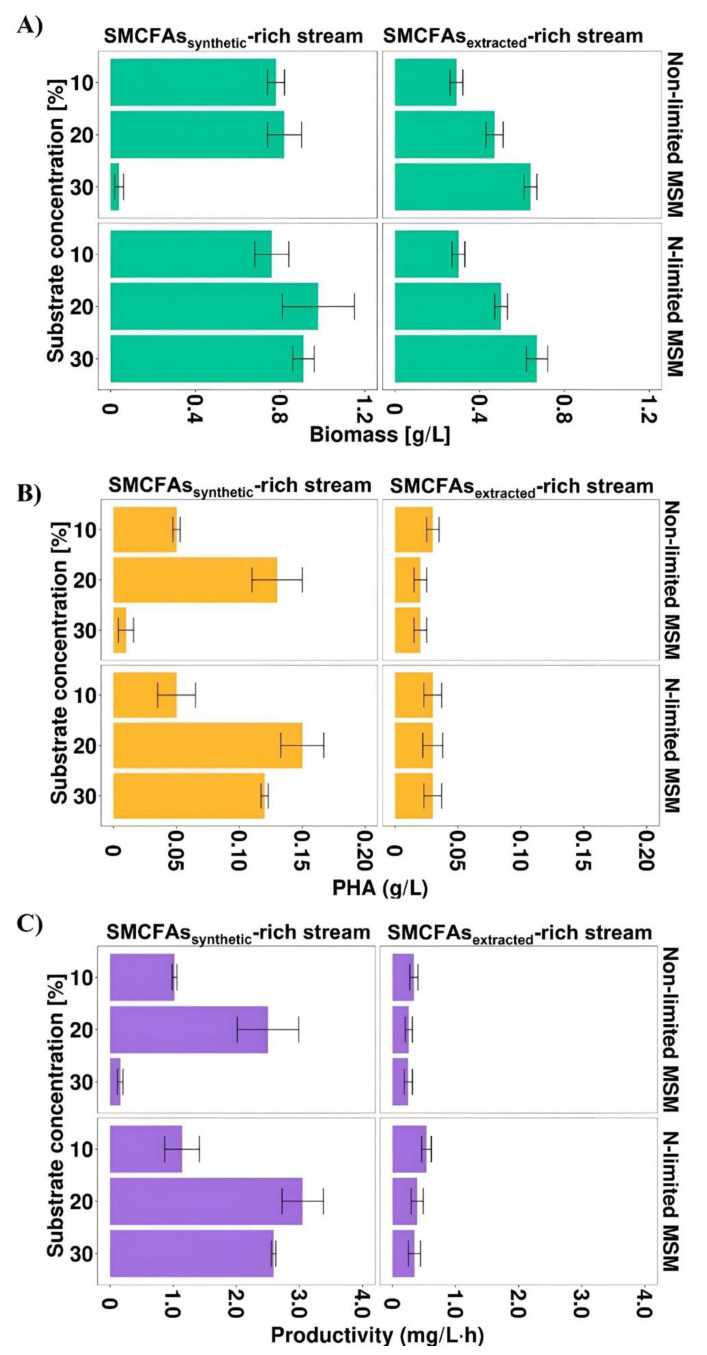
(**A**) Biomass concentration, (**B**) PHA concentration and (**C**) PHA productivity profiles of *Aeromonas* sp. AC_01 at 48 h of the cultivations supplemented with SMCFAs_synthetic_-rich stream and SMCFAs_extracted_-rich stream. Abbreviations: non-limited MSM, non-limited mineral salt medium; N-limited MSM, nitrogen limited mineral salt medium.

Most of the previous studies described individual SMCFAs as carbon sources towards PHA production. However, the effluent from whey wastewater anaerobic fermentation used in this study was complex and, except for SMCFAs, consisted of other compounds (lactic acid and ethanol). To investigate whether the non-SMCFAs compounds could have impact on *Aeromonas* sp. AC_01 growth and its PHA productivity, the simulated SMCFAs mixture was prepared identically with the SMCFAs components of the SMCFAs-rich stream. Due to the fact that the proper SMCFAs concentration is essential to achieve satisfying bacterial growth and consequently higher PHA yield, three different concentrations of the carbon sources were tested (10, 20, and 30% *v*/*v*). Figure 3A–C showed that biomass concentration, PHA concentration and PHA productivity were dependent on the amount of the carbon source used.

In all experimental variants the values of these parameters were higher in the cultivations with simulated SMCFAs-rich stream compared to SMCFAs-rich stream derived from anaerobic fermentation. The biomass value was the highest in the cultivation under both applied conditions supplemented with 30 g/L of the effluent indicating high tolerance of the *Aeromonas* sp. AC_01 to SMCFAs-rich stream. Nevertheless, the highest PHA concentration and PHA productivity was lower than that for the simulated SMCFAs mixture. The maximum PHA yield and efficiency was achieved with 10% of SMCFAs-rich stream, i.e., 0.03 g/L and 0.54 mg/L·h, respectively. It could suggest that there are inhibitors in the extracted SMCFAs mixture that due to its complex composition may adversely affect PHA biosynthesis process. Based on the chemical characterization, the main difference between the simulated SMCFAs solution and SMCFAs-rich stream was the presence of ethanol and lactic acid in the latter. Those components had inhibition effects on the *Aeromonas* sp. AC_01 growth and in a consequence on the PHA concentration and productivity. These results were inconsistent with a study of Obruca et al. [31] who proved that *Cupriavidus necator* exposed to ethanol at the beginning of the stationary phase intensified P(3HB) yields about 30%. Moreover, Martinez et al. [6] reported lower PHA productivity (0.022 mg/L·h) compared to the above-described data in the cultivation of *Cupriavidus necator* supplemented with olive mill wastewater effluent rich in acetic, propionic and butyric acid. However, the effluent consisted of not only different short chain fatty acids, but also polyphenols (1.2 g/L), N-NH_4_ (60 mg/L), proteins (1.56 g/L), and lipids (3.24 g/L). Furthermore, in our study, nitrogen-starvation conditions were beneficial to PHA production process using synthetic mixture, whereas in the culture supplemented with SMCFAs-rich stream PHA synthesis was on the same level.

### 3.4. GC-MS Analysis

The monomer composition of the accumulated PHAs varied with the carbon source and culture conditions used (Table 3). The highest synthesis efficiency of 3HV monomer was determined in the cultivation supplemented with valeric acid. It was previously confirmed that acids with an even number of carbon on the molecule are more favorable to the synthesis of 3HB, while acids with odd number of carbon on the molecule tends to produce 3HV [32]. To produce 3HB monomers only acetyl-CoA is required which is formed during metabolism of acids with an even number of carbon in the molecule. Whereas, to synthesize 3HV, bacteria need to produce acetyl-CoA and propionyl-CoA. The latter is produced from pyruvate and valeryl-CoA formed from valeric acid. Interestingly, when valeric acid was used as the only carbon source, PHA composed of uneven carbon numbered monomers (3-hydroxytridecanoic acid) was accumulated by *Aeromonas* sp. AC_01, with the highest content (23.6 mol%) detected in the PHAs extracted from the bacterial cells cultured on 1 g/L of valeric acid under non-limited conditions. Moreover, a concentration of 3HB molar fraction in the P(3HB-co-3HV-co-3HTriD-co-3HTD) copolyester seems to be dependent on the valerate concentration and culture condition. The highest 3HB content (32.9 mol%) was detected in the cultivation with 1 g/L of valerate under non-limited conditions. Except for 3HB, 3HV and 3HTriD monomers in the extracted copolymers 3HTD was also detected, however in trace amount (from 1.58 to 8.31 mol%). So far, no data has been reported about the ability of bacteria belong to *Aeromonas* genera to produce copolyester consisting of 3HB, 3HV, 3-HTriD and 3-HTD. Terpolyester P(3HB-co-3HV-co-3HHx) was reported to be accumulated by *Aeromonas hydrophila* 4AK4 cultivated on mixed carbon sources of lauric acid and valerate [25]. The authors confirmed that 3HV content was affected by valerate concentration added to the culture medium.

Furthermore, the 3HV monomers were also detected in biopolymers extracted from the tested bacterium grown with caproic acid but to a lesser extent (from 3.52 to 18.05 mol%) in comparison to that determined with valeric acid. Furthermore, when caproic acid was supplied 3HB monomer was also detected as a main constituent and trace amounts of 3HTD were found in all cultivations. It may be assumed that caproate or its intermediates involved in metabolism are less efficient substrates to produce monomers consisting of higher numbers of carbon. The same observations was made by Cerrone et al. [19] who cultivated *P. putida* strains with propionate and also observed an uneven carbon chain in extracted PHA. However, its content was lesser compared to that revealed with valeric acid.

When acetic acid and butyric acid were used as co-substrates, the synthesis of 3HB and 3HTD was observed. Along with the increase of acetic acid ratio in the mixture, the content of 3HB fraction in the copolymer decreased whereas the 3HTD content increased under both applied culture conditions. Furthermore, the data obtained from the cultivations with these single acids indicated that acetic acid supported the synthesis of 3HTD monomer whereas in the cultivation with butyric acid the 3HB monomers were detected and were dominant.

As can be seen in Table 3, the biopolyester composition in the cultivations with SMCFAs_synthetic_-rich stream depends on the carbon source concentration used. The obtained results proved that the analyzed strain showed a tendency to accumulate P(3HB-co-3HV-co-3HTD) terpolyester in the cultivations with the highest concentration of the simulated mixture. With decreasing concentration of this substrate, *Aeromonas* sp. AC_01 synthesized P(3HB-co-3HTD) copolymer with large amounts of 3HB and lesser content of 3HTD. Interestingly, when SMCFAs-rich stream was supplied, the monomeric composition of PHAs depends not only on the substrate concentration supplied but also on the culture condition used. The P(3HB-co-3HV-co-3HTD) terpolyester was detected only in the cultivation with 10% of SMCFAs-rich stream under N-limited condition.

### 3.5. PHAs Spectroscopic Analysis

**Figure 4:**
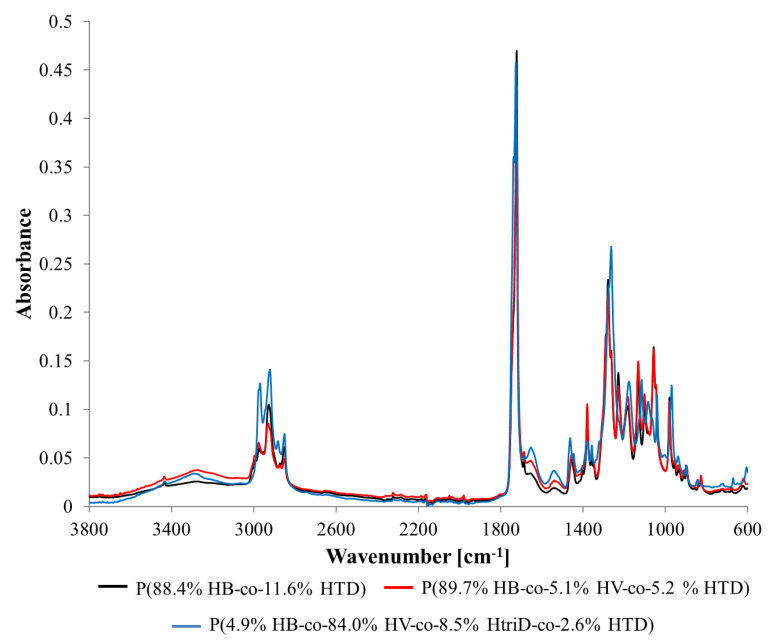
FTIR spectra of selected samples: (**black line**) P(88.4% HB-co-11.6% HTD) extracted from the culture supplemented with 10% SMCFAs_synthetic_-rich stream under nitrogen-limited condition (**red line**) P(89.7% HB-co-5.1% HV-co-5.2% HTD) extracted from the culture supplemented with 1g/L of caproic acid under non-limited condition, (**blue line**) P(4.9% HB-co-84.0% HV-co-8.5% HtriD-co 2.6% HTD) extracted from the culture supplemented with 2 g/L of valeric acid under nitrogen-limited condition.

FTIR spectra of selected representative samples recorded in the range from 4000 to 600 cm^−1^ are shown in Figure 4 and Supplementary Materials Figure S1. Due to the similar structure of individual 3HV, 3HtriD, and 3HTD units, the obtained spectra were similar regardless of the obtained monomeric composition of the polymer. Any observed differences in the absorbance of individual peaks resulted mainly from the characteristics of the sample, e.g., thickness.

Several bands have been observed that can be assigned to the functional groups characteristic for PHAs [33,34]. Table 4 presents the wavenumbers of individual peaks with associated functional groups and/or bonds. The values given in the table are averaged and the deviations for individual samples did not exceed a few units.

For some biopolymers (extracted from the cultivations with valeric acid, caproic acid, 0.4 HAc:1.6 HBu and 10% SMCFAs_synthetic_-rich stream under N-limited condition) a slight band with a maximum of ~3300 cm^−1^ was observed. It probably originates from terminal -OH groups present at the ends of the polymer chains. Their presence may suggest that extracted polymers have high number of oligomers. The band in the range of 3100–2700 cm^−1^ is associated with the presence of the -CH_3_ and -CH_2_ groups. The single peak band with a maximum of ~1723 cm^−1^ is derived from C=O stretching vibrations of the carboxyl groups present in macromolecules. In the spectra range from 1500 to 800 cm^−1^, which is a so-called fingerprint region, several bands with numerous peaks were recorded. Peaks ~1455, ~1380, ~980, ~900, and ~825 cm^−1^ are associated with the presence of the -CH_3_ and -CH_2_ groups, as well as C-C bonds which are representative of chemical structures of 3HB monomers [20]. Peaks ~1279, ~1229, ~1183, ~1133, ~1101, and ~1057 cm^−1^ are associated with bonds in the C-O-C region. Also, one nitrogen-related band at ~1539 cm^−1^ was identified in the extracted biomaterials that was related to the N-O asymmetrical stretching bond.

### 3.6. PHAs Thermal Properties

The thermal properties of PHAs are important for their processing and applications. Determination of the thermal parameters of the produced biopolymers allows for the selection of appropriate processing temperatures and determination of their characteristics at operating temperatures. Our results revealed that the type of the substrate and in a consequence monomeric composition of the extracted PHAs influenced their thermal properties (Table 5).

**Figure 5:**
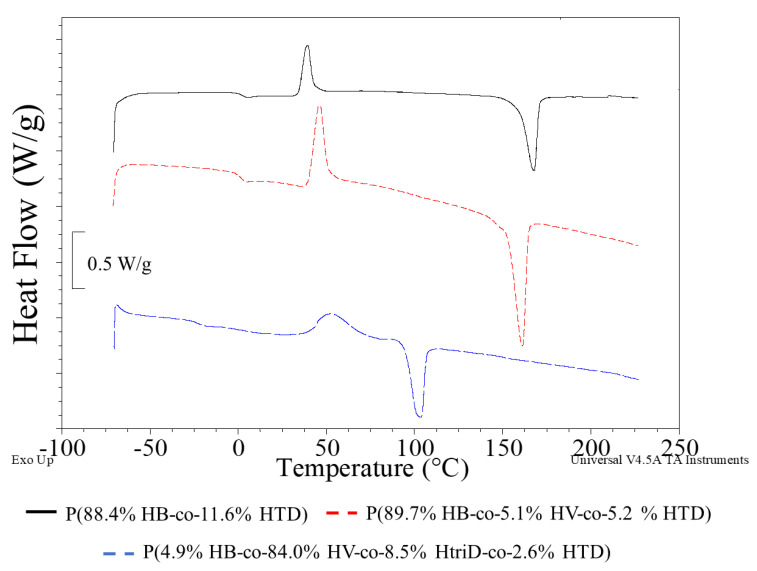
DSC curves of selected samples: (**black line**) P(88.4% HB-co-11.6% HTD) extracted from the culture supplemented with 10% SMCFAs_synthetic_-rich stream under nitrogen-limited condition (**red line**) P(89.7% HB-co-5.1% HV-co-5.2% HTD) extracted from the culture supplemented with 1g/L of caproic acid under non-limited condition, (**blue line**) P(4.9% HB-co-84.0% HV-co-8.5% HtriD-co 2.6% HTD) extracted from the culture supplemented with 2 g/L of valeric acid under nitrogen-limited condition.

Regardless of the monomeric composition of the macromolecule, phase changes related to glass transition, cold crystallization and melting were observed on the DSC curves of the tested polymers (Figure 5; Supplementary Materials Figure S2).

The glass transition temperature (T_g_) of pure P(3HB) was estimated to be 5 °C and its melting point (T_m_) was determined at the level of 170–180 °C [35,36]. In our study, the lowest T_g_ value was observed in the PHAs extracted from the cultivations supplemented with valeric acid under non-limited and N-limited condition. The 3HtriD monomer seems to affect the thermal properties of the extracted copolymers. The presence of this unit caused a decrease in T_g_ to −18.7 or −22.7 °C depending on the mer content (the higher content, the lower T_g_ values). Also, the T_m_ decreased compared to the other extracted biopolymers. The recorded T_m_ values of the P(3HB-co-3HV-co-3HtriD-co-3HTD) co-polymer were 104.4 or 103.3 °C (i.e., they were lower by more than 50 °C compared to the P(3HB-co-3HTD) and P(3HB-co-3HV-co-3HTD) copolymers). Furthermore, T_m_ values determined for the copolymers extracted from bacterial cells grown on SMCFAs-rich stream derived from acidogenic fermentation were slightly lower compared to the melting temperature for PHAs produced by *Aeromonas* sp. AC_01 cultured on synthetic SMCFAs-rich stream. However, these values were high enough to suggest that these biomaterials could be used in commercial purposes. It was proved that petrochemical derived polymers utilized for general purpose, engineering and high temperature specialty need to possess melting temperatures around 100, 150, and 300 °C, respectively [37].

**Figure 6:**
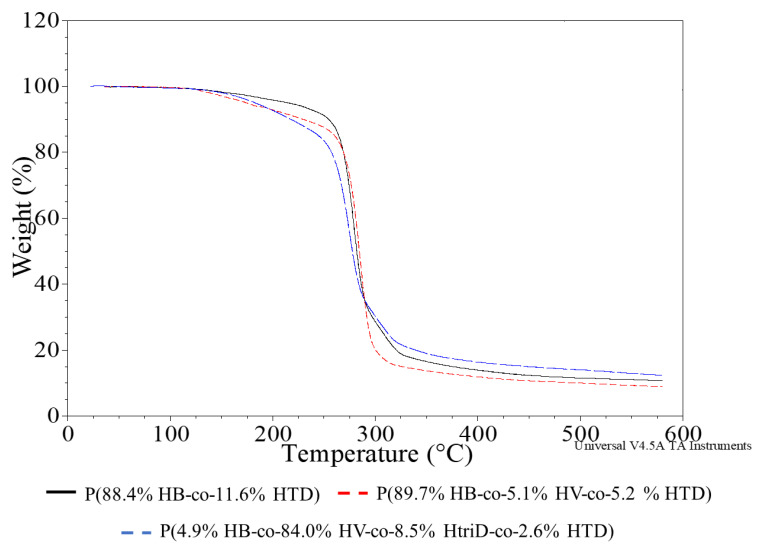
TGA thermograms of selected samples; (**black line**) P(88.4% HB-co-11.6% HTD) extracted from the culture supplemented with 10% SMCFAs_synthetic_-rich stream under nitrogen-limited condition (**red line**) P(89.7% HB-co-5.1% HV-co-5.2% HTD) extracted from the culture supplemented with 1g/L of caproic acid under non-limited condition, (**blue line**) P(4.9% HB-co-84.0% HV-co-8.5% HtriD-co 2.6% HTD) extracted from the culture supplemented with 2 g/L of valeric acid under nitrogen-limited condition.

TGA thermograms were recorded to evaluate the thermostability of the PHAs produced by *Aeromonas* sp. AC_01 (Figure 6, Supplementary Materials Figure S3).

The degradation temperature of PHAs, determined on the basis of the 5% weight loss temperature (T_5%_), ranges from 220 to 300 °C depending on the monomeric composition, with the most popular P(3HB) having a degradation temperature of about 225 °C [34,38]. The recorded T_5%_ values of the tested biopolymers ranged from 155.8 to 243.6 °C, with no clear relationship between their monomeric composition and T_5%_ value. The highest thermal stability was observed for the co-polymers extracted from the *Aeromonas* sp. AC_01 cells cultured on SMCFAs_synthetic_-rich stream. The other co-polymers were characterized to possess lower T_5%_ values than the typical degradation temperatures of PHAs. This was due to an additional minor degradation step ranging from 100 to 200 °C and preceding the main degradation process of the polymer. An additional degradation step may result from the presence of much lower molecular weight compounds in the polymer (non-polymer compounds or oligomers), which are left over from the polymer production process or are formed during polymer synthesis and are characterized by low degradation temperatures. However, FTIR studies indicate the latter, as polymers with higher T_5%_ values had no or a smaller peak from the terminal -OH groups. In the case of these co-polymers, no additional degradation stage was observed.

An additional parameter (i.e., onset temperature of the main stage of the sample decomposition (T_ons_)) was determined which did not take into account the decrease in the temperature value due to the first stage of degradation. Most of the polymers had very similar T_ons_, as the recorded values ranged from 261.3 to 272.3 °C. Therefore, it can be concluded that their thermal resistance is similar regardless of the obtained monomeric composition.

## 4. Conclusions

Our study confirmed that *Aeromonas* sp. AC_01 represents a highly promising candidate for the biotechnological production of PHA being able to utilize SMCFAs for the production of unique short-chain-length–medium-chain-length (scl-mcl)PHAs copolymers. Furthermore, we revealed that SMCFAs-rich stream was not toxic for *Aeromonas* sp. AC_01 and could support bacterial growth and PHA biosynthesis. The composition of SMCFAs could be regulated by the type of feedstock and operation conditions. In the SMCFAs-rich stream used in this study, butyric acid was the dominant product. The GC-MS and FTIR analyses confirmed that the monomeric composition of the extracted PHAs was dependent on the type of the carbon source used and its concentration. Our study proves that *Aeromonas* sp. AC_01 has the ability to change the repeat-units composition of the produced PHAs according to the culture conditions. Furthermore, the results of DSC and TG analyses revealed that the incorporation of 3HtriD monomer in the PHA structure lowered its melting temperature. All extracted PHAs possess high thermal stability which is beneficial taking into account the perspective of these biopolymers processing technology. However, to make this process more efficient, optimization is needed. The PHA productivity should be improved by feeding adjustment and optimization of nutrients and oxygen as well as initial inoculum in the fermentation system. Moreover, the obtained data should be supplemented with additional material analysis especially mechanical properties of the biopolymers that could provide information about their usefulness in particular application areas.

## Figures and Tables

**Table 1:** Composition of SMCFAs_extracted_ -rich stream used in this study.

Components	[g/L]	[%]
Acetic acid	2.85	10.61
Butyric acid	9.86	36.70
Valeric acid	0.16	0.60
Caproic acid	3.05	11.35
Lactic acid	9.31	34.65
Ethanol	1.64	6.10

**Table 2:** Biomass concentration and PHA production from individual short and medium chain fatty acids by *Aeromonas* sp. AC_01.

Medium		Substrate	Substrate Concentration [g/L]	Biomass [g/L]			PHA [% of CDM]			pH Value		
				24 h	48 h	72 h	24 h	48 h	72 h	24 h	48 h	72 h
**Non-limited MSM**	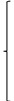	Acetic acid	1	0.61 ± 0.07	0.55 ± 0.14	1.48 ± 0.08	4.76 ± 0.05	1.11 ± 0.12	1.79 ± 0.10	7.66 ± 0.00	7.52 ± 0.01	7.75 ± 0.01
			2	0.85 ± 0.04	1.87 ± 0.09	1.73 ± 0.16	4.95 ± 0.05	1.27 ± 0.07	1.80 ± 0.24	7.54 ± 0.01	7.42 ± 0.00	7.74 ± 0.00
			3	0.51 ± 0.04	1.89 ± 0.24	1.99 ± 0.07	7.60 ± 0.84	1.41 ± 0.02	1.94 ± 0.39	7.43 ± 0.00	7.31 ± 0.01	7.68 ± 0.01
		Butyric acid	1	0.39 ± 0.04	0.67 ± 0.004	1.56 ± 0.07	14.90 ± 1.07	9.51 ± 0.63	2.53 ± 0.09	6.65 ± 0.01	6.68 ± 0.01	6.93 ± 0.01
			2	0.37 ± 0.08	0.27 ± 0.03	1.09 ± 0.09	13.68 ± 0.44	17.25 ± 0.82	5.02 ± 0.44	6.56 ± 0.01	6.52 ± 0.01	6.86 ± 0.01
			3	0.18 ± 0.04	0.25 ± 0.03	0.54 ± 0.13	13.15 ± 0.75	13.48 ± 0.34	13.04 ± 1.76	6.44 ± 0.01	6.51 ± 0.01	6.81 ± 0.01
		Valeric acid	1	0.39 ± 0.01	0.79 ± 0.11	0.91 ± 0.05	8.57 ± 0.42	3.51 ± 0.37	4.81 ± 0.61	6.65 ± 0.02	6.47 ± 0.01	6.83 ± 0.02
			2	0.24 ± 0.03	0.30 ± 0.09	0.39 ± 0.03	10.08 ± 1.24	5.58 ± 0.82	8.20 ± 0.10	6.56 ± 0.01	6.34 ± 0.04	6.65 ± 0.04
			3	0.24 ± 0.04	0.29 ± 0.00	0.45 ± 0.03	10.07 ± 1.01	3.09 ± 1.06	7.24 ± 0.30	6.41 ± 0.01	6.32 ± 0.02	6.45 ± 0.02
		Caproic acid	1	0.15 ± 0.02	0.53 ± 0.02	0.94 ± 0.07	23.87 ± 2.90	6.79 ± 0.69	5.59 ± 1.08	6.53 ± 0.10	6.48 ± 0.08	6.57 ± 0.08
			2	0.15 ± 0.02	0.37 ± 0.06	0.30 ± 0.03	23.36 ± 1.63	4.83 ± 0.24	10.70 ± 0.61	6.54 ± 0.02	6.36 ± 0.08	6.51 ± 0.16
			3	0.04 ± 0.01	0.28 ± 0.05	0.26 ± 0.06	28.17 ± 0.56	5.90 ± 0.46	6.10 ± 0.76	6.44 ± 0.04	6.23 ± 0.03	6.44 ± 0.15
**N-limited MSM**	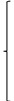	Acetic acid	1	0.37 ± 0.02	0.46 ± 0.06	0.82 ± 0.07	5.86 ± 1.22	15.35 ± 0.43	7.10 ± 0.68	7.65 ± 0.01	7.55 ± 0.01	7.56 ± 0.01
			2	0.42 ± 0.02	0.82 ± 0.04	1.63 ± 0.21	5.66 ± 0.55	8.73 ± 0.51	3.45 ± 0.01	7.59 ± 0.01	7.46 ± 0.05	7.52 ± 0.01
			3	0.48 ± 0.04	0.99 ± 0.12	1.63 ± 0.25	6.63 ± 0.76	5.74 ± 0.06	3.37 ± 0.72	7.48 ± 0.01	7.43 ± 0.01	7.45 ± 0.02
		Butyric acid	1	0.24 ± 0.01	0.46 ± 0.10	0.56 ± 0.02	14.23 ± 1.88	17.93 ± 0.73	7.54 ± 1.06	6.65 ± 0.00	6.52 ± 0.01	6.67 ± 0.01
			2	0.24 ± 0.01	0.27 ± 0.03	1.00 ± 0.06	14.93 ± 1.65	17.67 ± 1.00	8.23 ± 0.01	6.65 ± 0.01	6.51 ± 0.01	6.72 ± 0.01
			3	0.13 ± 0.01	0.19 ± 0.03	0.18 ± 0.01	18.56 ± 0.54	13.19 ± 0.98	17.91 ± 0.16	6.64 ± 0.02	6.52 ± 0.01	6.67 ± 0.01
		Valeric acid	1	0.42 ± 0.06	0.60 ± 0.10	0.67 ± 0.13	11.01 ± 0.54	4.80 ± 0.89	3.47 ± 0.23	6.73 ± 0.01	6.52 ± 0.02	6.76 ± 0.11
			2	0.26 ± 0.08	0.28 ± 0.01	0.85 ± 0.02	13.94 ± 2.04	8.80 ± 0.57	12.38 ± 0.16	6.65 ± 0.04	6.53 ± 0.03	6.83 ± 0.03
			3	0.19 ± 0.05	0.29 ± 0.01	0.31 ± 0.03	12.97 ± 0.94	10.34 ± 0.48	12.52 ± 0.16	6.62 ± 0.02	6.53 ± 0.03	6.75 ± 0.03
		Caproic acid	1	0.22 ± 0.05	0.57 ± 0.04	0.70 ± 0.01	16.83 ± 0.24	9.38 ± 0.86	4.59 ± 0.36	6.72 ± 0.03	6.62 ± 0.14	6.91 ± 0.03
			2	0.10 ± 0.01	0.17 ± 0.04	0.20 ± 0.01	9.21 ± 0.36	19.84 ± 0.28	18.31 ± 0.81	6.70 ± 0.02	6.52 ± 0.02	6.80 ± 0.04
			3	0.13 ± 0.00	0.15 ± 0.02	0.18 ± 0.03	9.61 ± 0.36	20.38 ± 0.92	13.36 ± 0.61	6.63 ± 0.01	6.41 ± 0.02	6.75 ± 0.03

Non-limited MSM, non-limited mineral salt medium; N-limited MSM, nitrogen limited mineral salt medium; PHA, polyhydroxyalkanoates; CDM, cell dry mass.

**Table 3:** Monomeric compositions of PHAs extracted from *Aeromonas* sp. AC_01 cells.

Carbon Source	Substrate Concentration [g/L; % *]	PHA Composition [mol%]			
		3HB	3HV	3HtriD	3HTD
**Non-limited MSM**					
Acetic acid	1	nd	nd	nd	100.0 ± 0.0
	2	nd	nd	nd	100.0 ± 0.0
	3	nd	nd	nd	100.0 ± 0.0
Butyric acid	1	83.4 ± 0.9	nd	nd	16.6 ± 0.9
	2	95.6 ± 0.3	nd	nd	4.4 ± 0.3
	3	93.3 ± 0.6	nd	nd	6.7 ± 0.6
Valeric acid	1	32.9 ± 0.3	35.1 ± 0.4	23.7 ± 0.1	8.3 ± 0.6
	2	4.6 ± 0.1	88.6 ± 0.0	5.1 ± 0.1	1.6 ± 0.0
	3	6.2 ± 0.2	87.1 ± 0.0	2.9 ± 0.2	3.9 ± 0.0
Caproic acid	1	89.6 ± 0.2	5.2 ± 0.1	nd	5.2 ± 0.1
	2	92.2 ± 0.5	3.5 ± 0.1	nd	4.3 ± 0.4
	3	92.2 ± 0.4	3.5 ± 0.4	nd	4.3 ± 0.0
Acetic acid:Butyric acid	0.4:1.6	92.0 ± 0.0	nd	nd	8.0 ± 0.0
	0.8:1.2	80.8 ± 0.5	nd	nd	19.3 ± 0.5
	1.2:0.8	77.1 ± 0.0	nd	nd	22.9 ± 0.0
	1.6:0.4	77.1 ± 0.7	nd	nd	22.9 ± 0.7
SMCFAs_synthetic_-rich stream	10 *	74.5 ± 0.5	nd	nd	25.5 ± 0.5
	20 *	95.5 ± 0.1	nd	nd	4.5 ± 0.1
	30 *	94.7 ± 0.1	0.9 ± 0.0	nd	4.4 ± 0.1
SMCFAs_extracted_-rich stream	10 *	95.6 ± 0.0	nd	nd	4.4 ± 0.0
	20 *	100.0 ± 0.0	nd	nd	nd
	30 *	71.7 ± 3.1	nd	nd	28.3 ± 3.1
**N-limited MSM**					
Acetic acid	1	48.9 ± 0.7	nd	nd	51.1 ± 0.7
	2	nd	nd	nd	100.0 ± 0.0
	3	nd	nd	nd	100.0 ± 0.0
Butyric acid	1	92.2 ± 0.2	nd	nd	7.8 ± 0.2
	2	94.6 ± 0.1	nd	nd	5.4 ± 0.1
	3	93.6 ± 0.2	nd	nd	6.4 ± 0.2
Valeric acid	1	7.8 ± 0.1	73.4 ± 0.1	13.8 ± 0.1	5.1 ± 0.0
	2	4.9 ± 0.0	83.9 ± 0.0	8.5 ± 0.0	2.6 ± 0.0
	3	2.8 ± 0.1	92.9 ± 0.1	2.8 ± 0.1	1.6 ± 0.0
Caproic acid	1	80.7 ± 0.2	nd	nd	19.3 ± 0.2
	2	82.3 ± 0.1	13.4 ± 0.1	nd	4.3 ± 0.0
	3	78.7 ± 0.0	18.1 ± 0.0	nd	3.2 ± 0.0
Acetic acid:Butyric acid	0.4:1.6	92.7 ± 0.2	nd	nd	7.3 ± 0.2
	0.8:1.2	86.1 ± 0.0	nd	nd	13.9 ± 0.0
	1.2:0.8	77.8 ± 0.3	nd	nd	22.2 ± 0.4
	1.6:0.4	65.4 ± 0.5	nd	nd	34.6 ± 0.5
SMCFAs_synthetic_-rich stream	10 *	88.4 ± 0.1	nd	nd	11.6 ± 0.1
	20 *	94.9 ± 0.2	0.6 ± 0.0	nd	4.5 ± 0.2
	30 *	90.6 ± 0.0	5.5 ± 0.0	nd	3.9 ± 0.1
SMCFAs_extracted_-rich stream	10 *	90.4 ± 0.2	2.5 ± 0.1	nd	7.1 ± 0.1
	20 *	90.3 ± 2.0	nd	nd	9.7 ± 2.0
	30 *	100.0 ± 0.0	nd	nd	nd

nd, not detected; 3HB, 3-hydroxybutyrate; 3HV, 3-hydroxyvalerate; 3HtriD, 3-hydroxytridecanoate; 3HTD, 3-hydroxytetradecanoate; non-limited MSM, non-limited mineral salt medium; N-limited MSM, nitrogen limited mineral salt medium; SMCFAs, short and medium chain fatty acids; SMCFAs_synthetic_-rich stream, a mixture of synthetic SMCFAs to simulate a real SMCFAs-rich stream; SMCFAs_extracted_-rich stream, a mixture of SMCFAs received from the acidogenic anaerobic mixed culture fermentation of acid whey. *—an unit of the concentration of SMCFA_Ssynthetic_-rich stream and SMCFA_Sextracted_-rich stream.

**Table 4:** Functional group and its quantified frequencies of extracted PHAs.

Wavenumber [cm^−1^]	Assignment
~3300	-OH, terminal
~2976	CH_3_, asymmetrical stretching
~2928	CH_2_, C-H asymmetrical stretching
~2852	CH_3_, C-H symmetrical stretching
~1723	C=O, stretching
~1658	C-O, stretching
~1539	N-O asymmetrical stretching
~1455	CH_3,_ C-H asymmetrical scissoring
~1380	CH_3,_ C-H symmetrical scissoring
~1279	C-O-C, asymmetrical stretching
~1229	C-O-C, asymmetrical stretching
~1183	C-O, asymmetrical stretching
~1133	C-O-C
~1101	C-O-C
~1057	C-O, asymmetrical stretching
~980	C-C, stretching
~900	CH_2_, rocking
~825	CH_3_, rocking

**Table 5:** Differential scanning calorimetry (DSC) and thermogravimetry (TG) results of the produced PHAs extracted from *Aeromonas* sp. AC_01 cells grown on butyric acid, valeric acid, caproic acid, a mixture of acetic and butyric acid, SMCFAs_synthetic_-rich stream and SMCFAs_extracted_-rich stream.

Substrate and Culture Condition	PHA Composition	DSC					TGA	
		T_g_ [°C]	T_cc_ [°C]	ΔH_cc_ [W/g]	T_m_ [°C]	ΔH_m_ [W/g]	T_5%_ [°C]	T_ons_ [°C]
Butyric acid, non-limited MSM	P(84.1% HB-co-15.9% HTD)	−0.3	39.1	19.1	165.5	44.4	155.8	269.8
Butyric acid, N-limited MSM	P(92.1% HB-co-7.9% HTD)	2.6	37.7	23.7	172.2	41.1	226.6	267.5
Valeric acid, non-limited MSM	P(4.7%HB-co-88.6% HV-co-5.1% HtriD-co-1.6% HTD)	−18.7	58.3	28.1	104.4	34.6	182.5	266.6
Valeric acid, N-limited MSM	P(4.9% HB-co-84.0% HV-co-8.5% HtriD-co-2.6% HTD)	−22.7	52.8	26.9	103.4	33.0	183.1	261.3
Caproic acid, non-limited MSM	P(89.7% HB-co-5.1% HV-co-5.2% HTD)	1.8	46.4	43.9	161.0	56.4	174.8	272.3
Caproic acid, N-limited MSM	P(80.8% HB-co-19.2% HTD)	0.7	40.8	11	161.9	27.4	183.5	265.5
Acetic acid:Butyric acid, non-limited MSM	P(92.0% HB-co-8.0% HTD)	2.6	41.0	26.9	172.8	78.1	183.8	271.5
Acetic acid:Butyric acid, N-limited MSM	P(93.0% HB-co-7.0% HTD)	1.7	51.7	39.8	166.9	57.7	181.4	267.4
20% SMCFAs_synthetic_-rich stream, non-limited MSM	P(95.5% HB-co-4.5% HTD)	3.3	47.3	13.5	167.3	61.7	225.2	269.7
20% SMCFAs_synthetic_-rich stream, N-limited MSM	P(94.7% HB-co-0.6% HV-co-4.7% HTD)	2.9	42.2	5.6	168.2	55.1	243.6	267.7
10% SMCFAs_synthetic_-rich stream, N-limited MSM	P(88.4% HB-co-11.6% HTD)	2.6	39.2	15.7	167.8	32.8	215.7	266.4
30% SMCFAs_synthetic_-rich stream, N-limited MSM	P(90.6% HB-co-5.5% HV-co-3.9% HTD)	2.0	46.7	14.4	156.6	29.0	235.7	266.2
10% SMCFAs_extracted_-rich stream, non-limited MSM	P(95.6% HB-co-4.4% HTD)	0.2	65.4	16.3	151.0	21.9	168.1	261.4
10% SMCFAs_extracted_-rich stream, N-limited MSM	P(90.6% HB-co-2.4% HV-co-7.0% HTD)	2.7	53.4	40.2	155.9	56.2	174.2	262.3

T_g_, glass transition temperature; T_cc_, cold crystallization temperature; ΔH_cc_, change in enthalpy of cold crystallization; T_m_, melting point; ΔH_m_, change in enthalpy of melting process; T_5%_, 5% mass loss temperature; T_ons_, mass loss onset temperature; non-limited MSM, non-limited mineral salt medium; N-limited MSM, nitrogen limited mineral salt medium; SMCFAs, short and medium chain fatty acids; SMCFAs_synthetic_-rich stream, a mixture of synthetic SMCFAs to simulate a real SMCFAs-rich stream; SMCFAs_extracted_-rich stream, a mixture of SMCFAs received from the acidogenic anaerobic mixed culture fermentation of acid whey.

## Data Availability

Data available upon request.

## Supplementary Materials

The following supporting information can be downloaded at: https://www.mdpi.com/article/10.3390/ma15134482/s1.
