# Supplementary material for: Conversion of Short and Medium Chain Fatty Acids into Novel Polyhydroxyalkanoates Copolymers by Aeromonas sp. AC_01
Source: Materials (Basel). 2022 Jun 25;15(13):4482. doi: 10.3390/ma15134482 (PMC9267140; doi:10.3390/ma15134482)
Supplement: Supplementary file 1 [file materials-15-04482-s001.zip › materials-1753321-supplementary.pdf]

Supplementary material

Conversion of short and medium chain fatty acids into novel polyhydroxyalkanoates copolymers by *Aeromonas* sp. AC_01

Karolina Szacherska[1], Krzysztof Moraczewski[2], Sylwester Czaplicki[3], Piotr Oleskowicz-Popiel[4], Justyna Mozejko-Ciesielska[1*]

[1]Department of Microbiology and Mycology, Faculty of Biology and Biotechnology, University of Warmia and Mazury in Olsztyn, 10-719 Olsztyn, Poland.

[2]Institute of Materials Engineering, Kazimierz Wielki University, 85-064 Bydgoszcz, Poland.

[3]Department of Plant Food Chemistry and Processing, Faculty of Food Sciences, University of Warmia and Mazury in Olsztyn, Pl. Cieszyński 1, 10-726 Olsztyn, Poland.

[4]Water Supply and Bioeconomy Division, Faculty of Environmental Engineering and Energy, Poznan University of Technology, 60-965 Poznan, Poland.

*Corresponding author: Justyna Mozejko-Ciesielska, Department of Microbiology and Mycology, Faculty of Biology and Biotechnology, University of Warmia and Mazury in Olsztyn, Oczapowskiego 1A, 10-719 Olsztyn, e-mail: justyna.mozejko@uwm.edu.pl, phone: (+48) (89) 5234234

Figure S1: FTIR spectra from the analysis of PHA extracted from *Aeromonas* sp. AC_01 grown in the: non-limited medium supplemented with 1 g/L butyric acid (A), nitrogen-limited medium supplemented with 1 g/L butyric acid (B), non-limited medium supplemented with 2 g/L valeric acid (C), nitrogen-limited medium supplemented with 1 g/L caproic acid (D), non-limited medium supplemented with 0.4 g/L acetic acid : 1.6 g/L butyric acid (E), nitrogen-limited medium supplemented with 0.4 Acetic acid : 1.6 Butyric acid (F), non-limited medium supplemented with 20% $SMCFAs_{synthetic}$-rich stream (G), nitrogen-limited medium supplemented with 20% $SMCFAs_{synthetic}$-rich stream (H), nitrogen-limited medium supplemented with 30% $SMCFAs_{synthetic}$-rich stream (I), non-limited medium supplemented with 10% $SMCFAs_{extracted}$-rich stream (J), nitrogen-limited medium supplemented with 10% $SMCFAs_{extracted}$-rich stream (K).

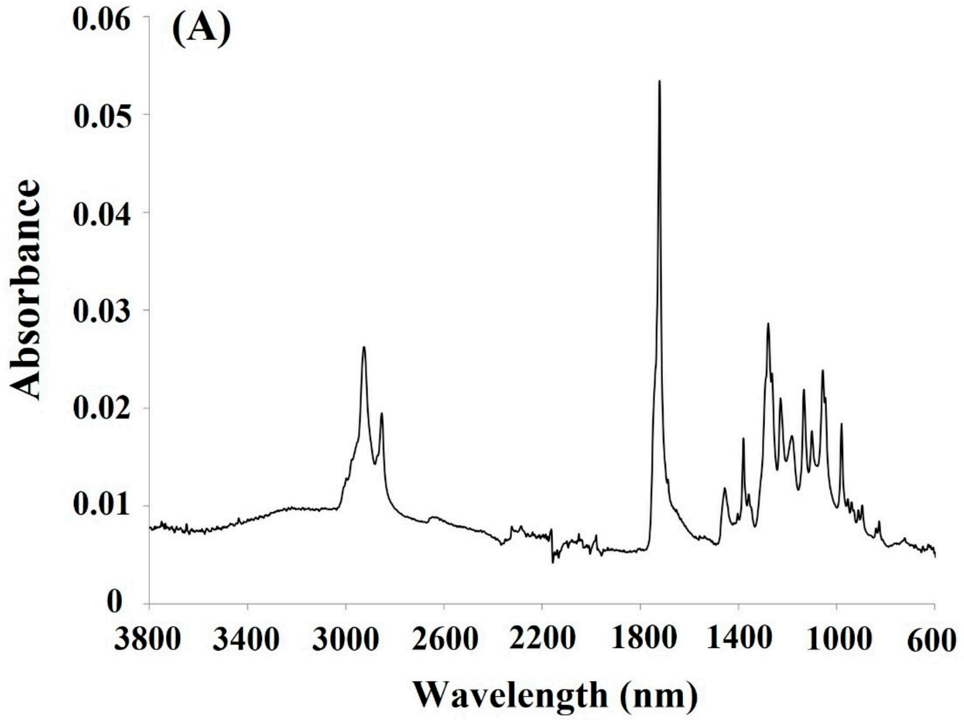

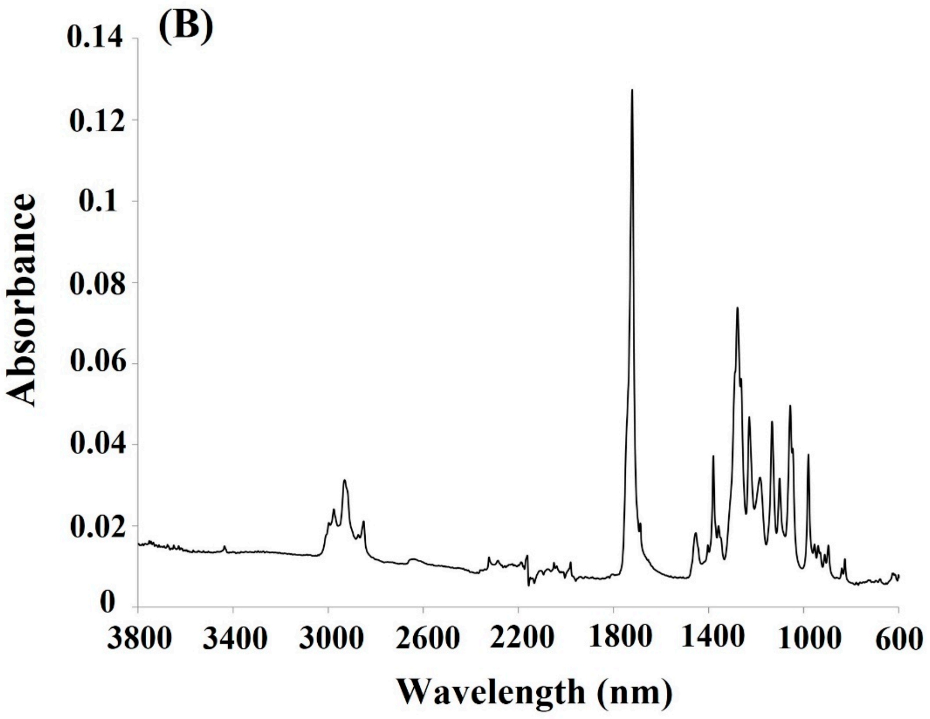
(B)
0.14
0.12
0.1
0.08
0.06
0.04
0.02
0
Absorbance
3800
3400
3000
2600
2200
1800
1400
1000
600
Wavelength (nm)

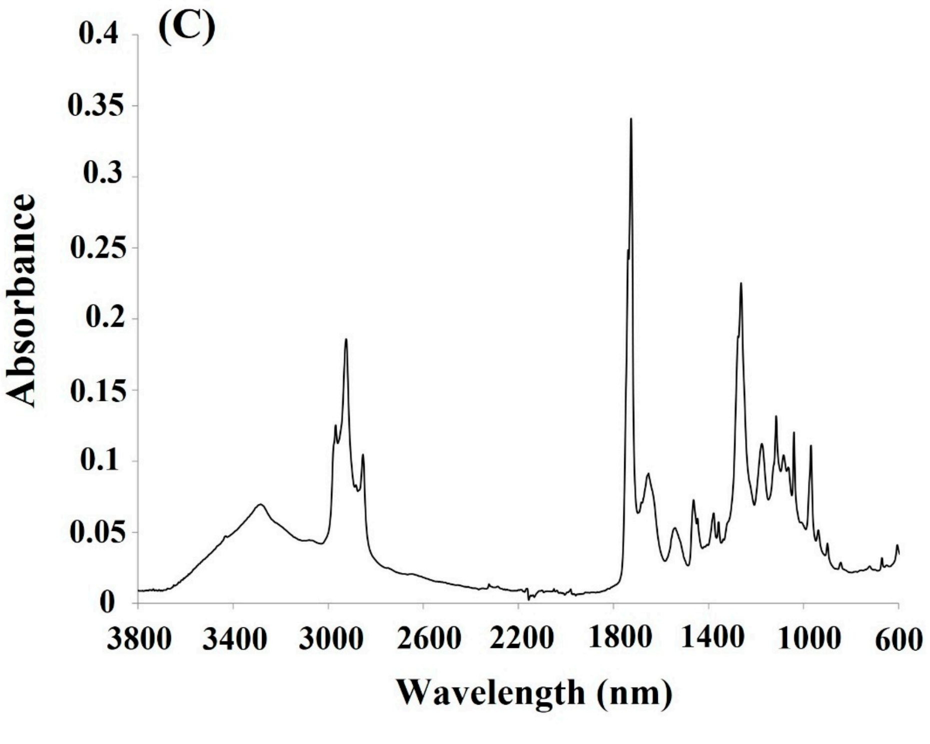
(C)
0.4
0.35
0.3
0.25
0.2
0.15
0.1
0.05
0
Absorbance
3800
3400
3000
2600
2200
1800
1400
1000
600
Wavelength (nm)

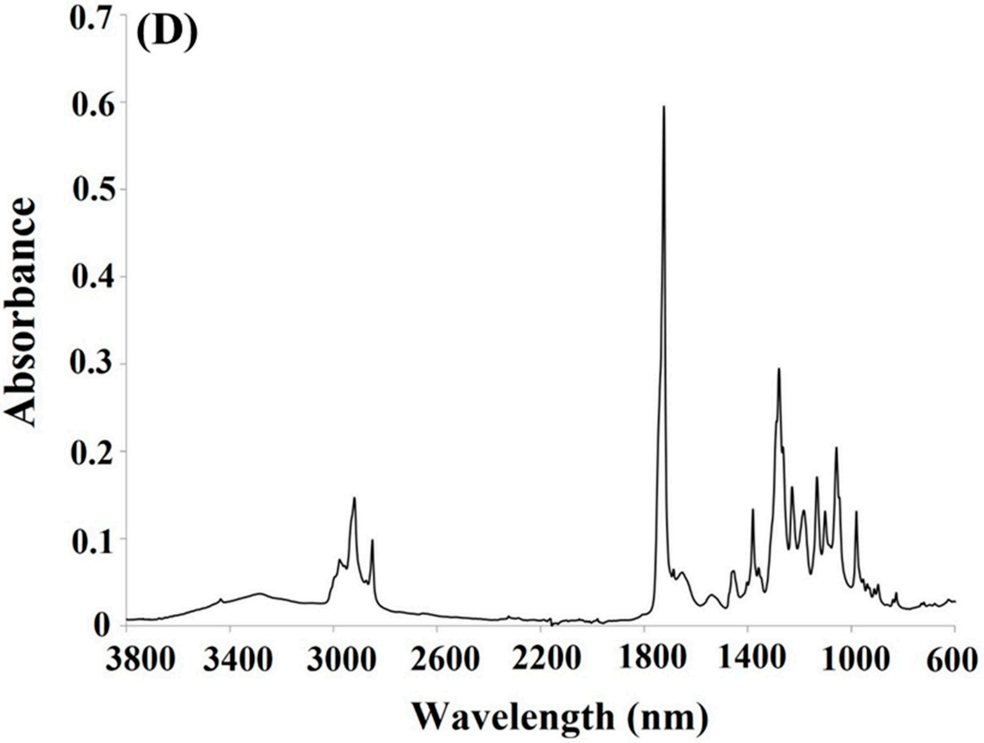
(D)
0.7
0.6
0.5
0.4
0.3
0.2
0.1
0
Absorbance
3800
3400
3000
2600
2200
1800
1400
1000
600
Wavelength (nm)

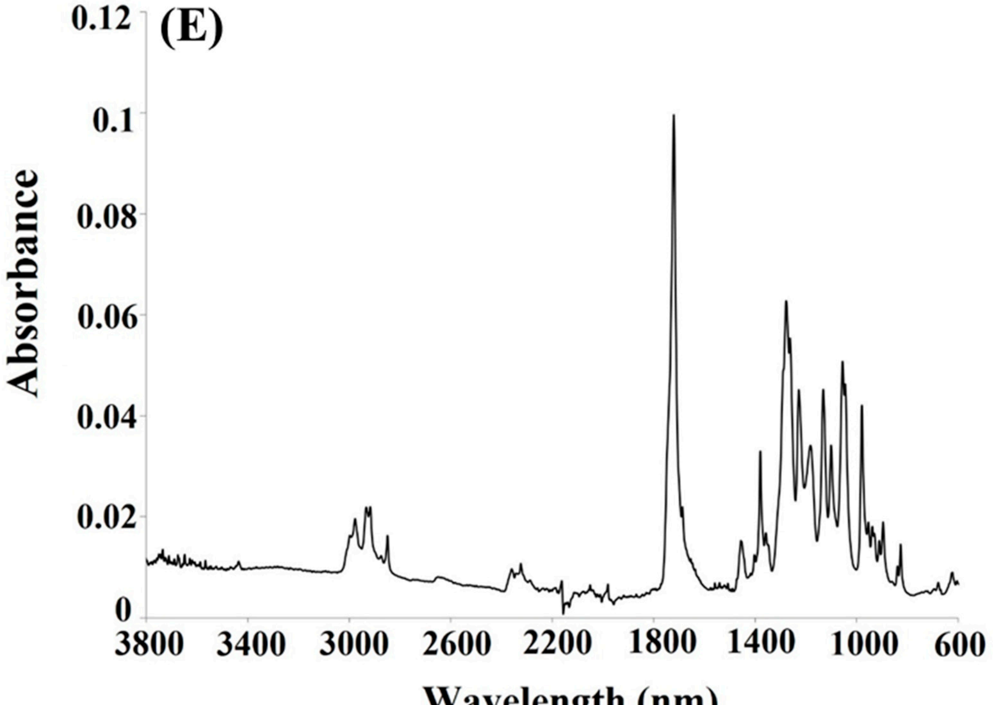
(E)
0.12
0.1
0.08
0.06
0.04
0.02
0
Absorbance
3800
3400
3000
2600
2200
1800
1400
1000
600
Wavelength (nm)

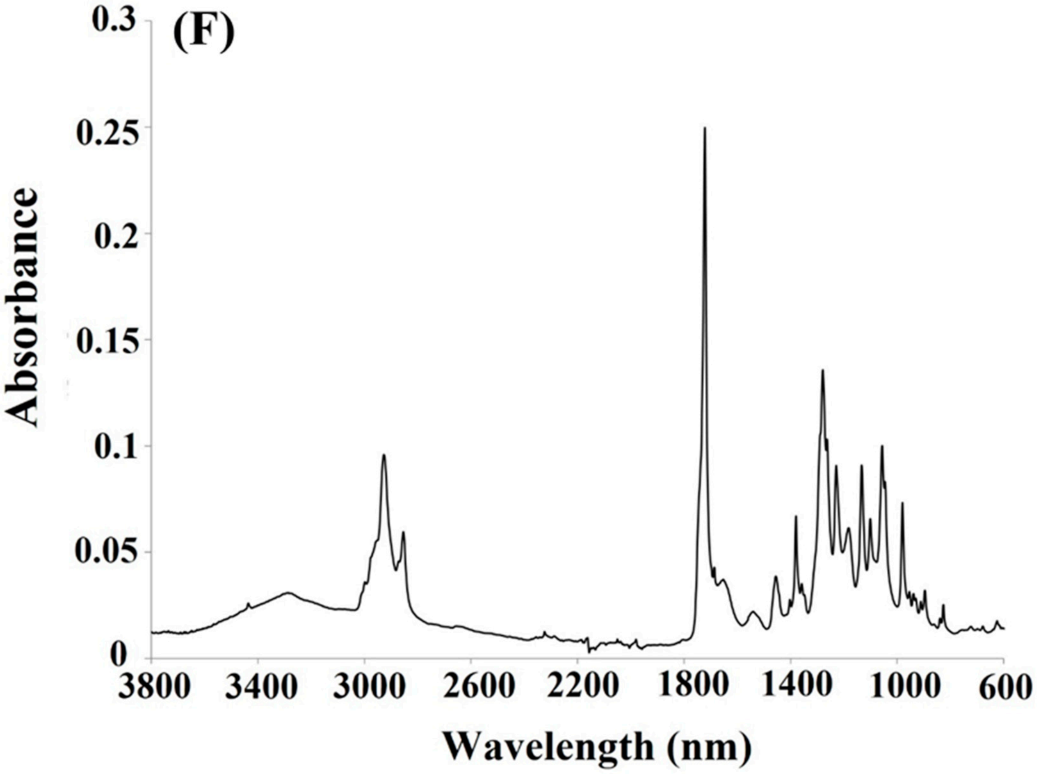
(F)
Absorbance
0.3
0.25
0.2
0.15
0.1
0.05
0
3800
3400
3000
2600
2200
1800
1400
1000
600
Wavelength (nm)

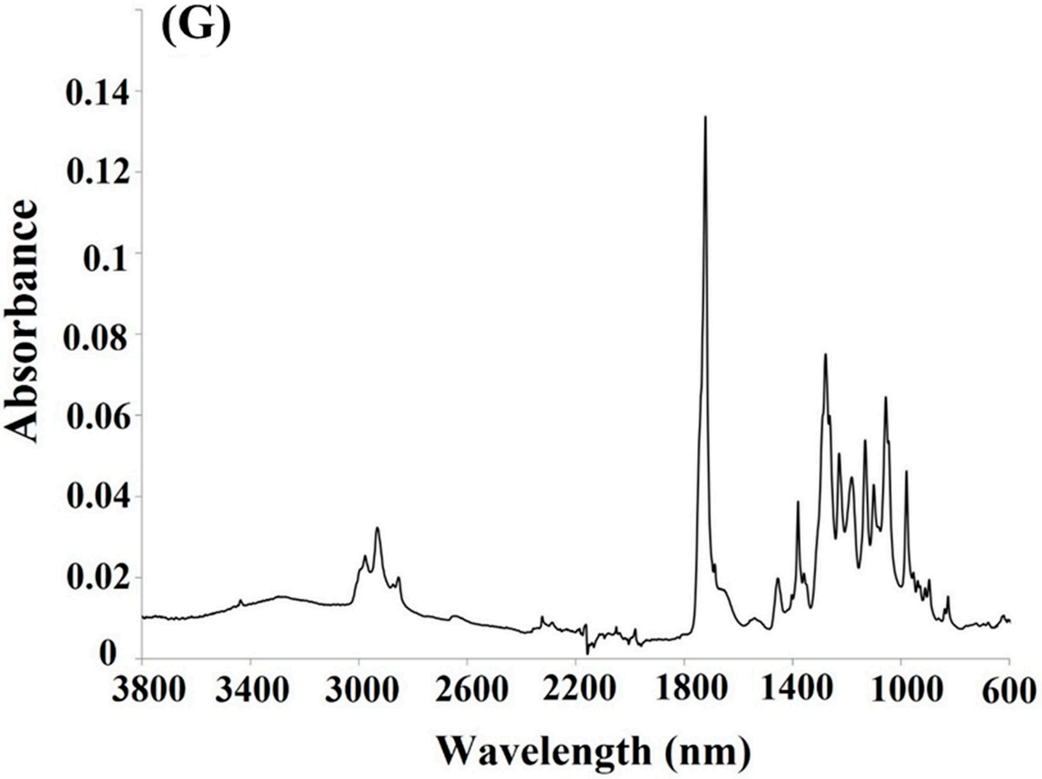
(G)
Absorbance
0.14
0.12
0.1
0.08
0.06
0.04
0.02
0
3800
3400
3000
2600
2200
1800
1400
1000
600
Wavelength (nm)

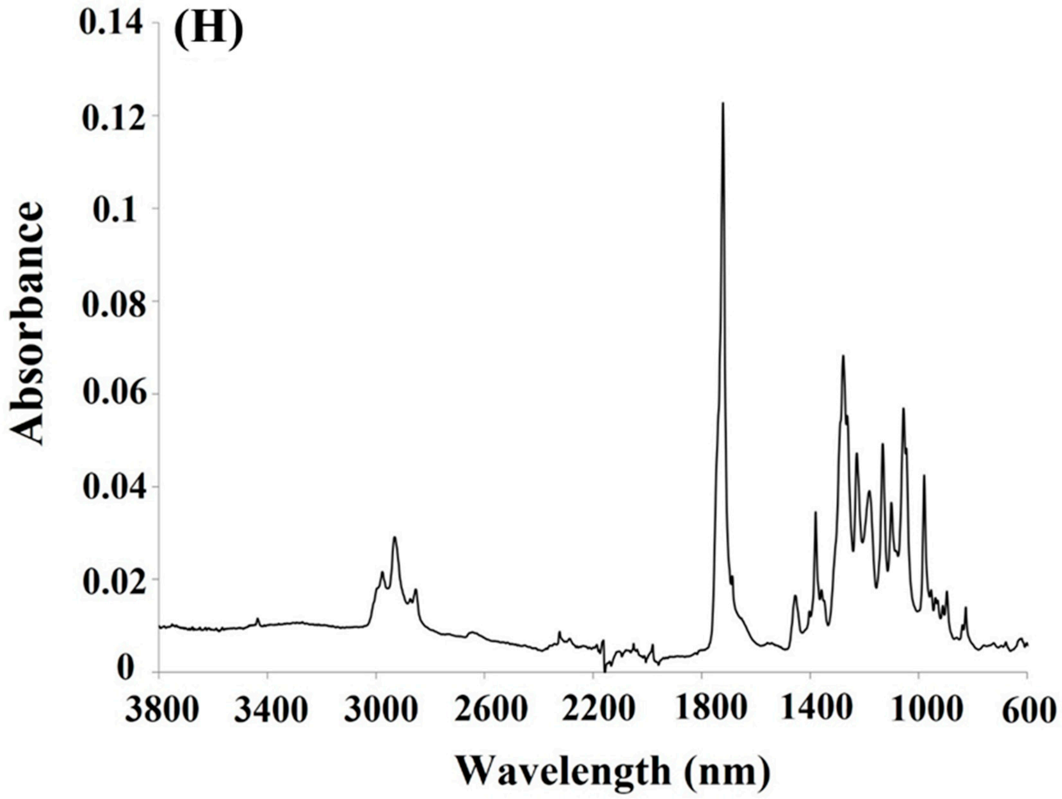
(H)
0.14
0.12
0.1
0.08
0.06
0.04
0.02
0
Absorbance
3800 3400 3000 2600 2200 1800 1400 1000 600
Wavelength (nm)

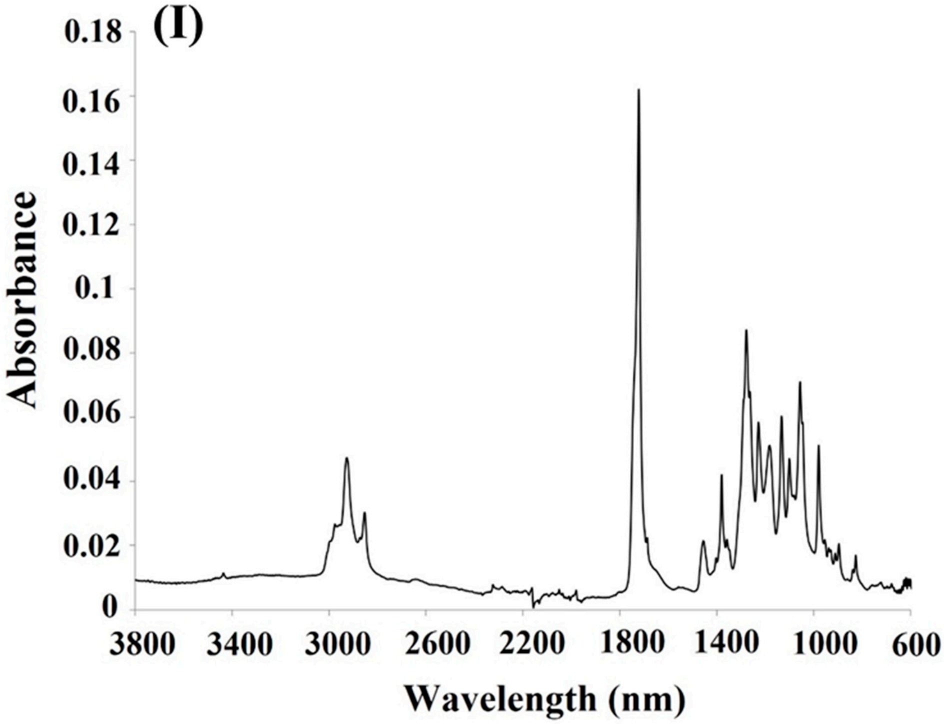
(I)
0.18
0.16
0.14
0.12
0.1
0.08
0.06
0.04
0.02
0
Absorbance
3800 3400 3000 2600 2200 1800 1400 1000 600
Wavelength (nm)

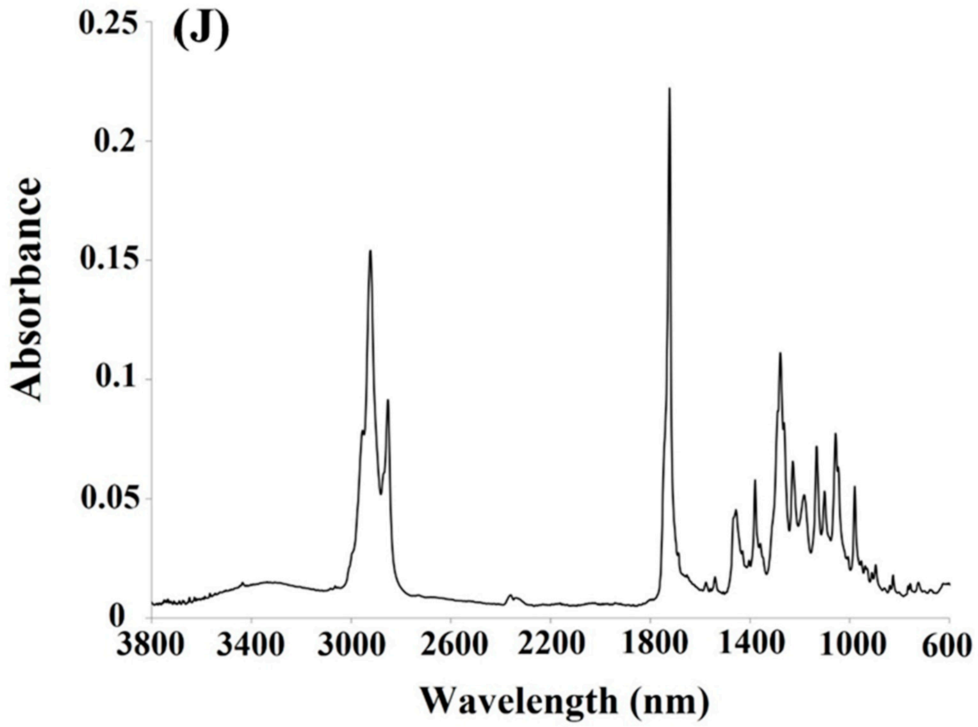
(J)
Absorbance
0.25
0.2
0.15
0.1
0.05
0
3800
3400
3000
2600
2200
1800
1400
1000
600
Wavelength (nm)

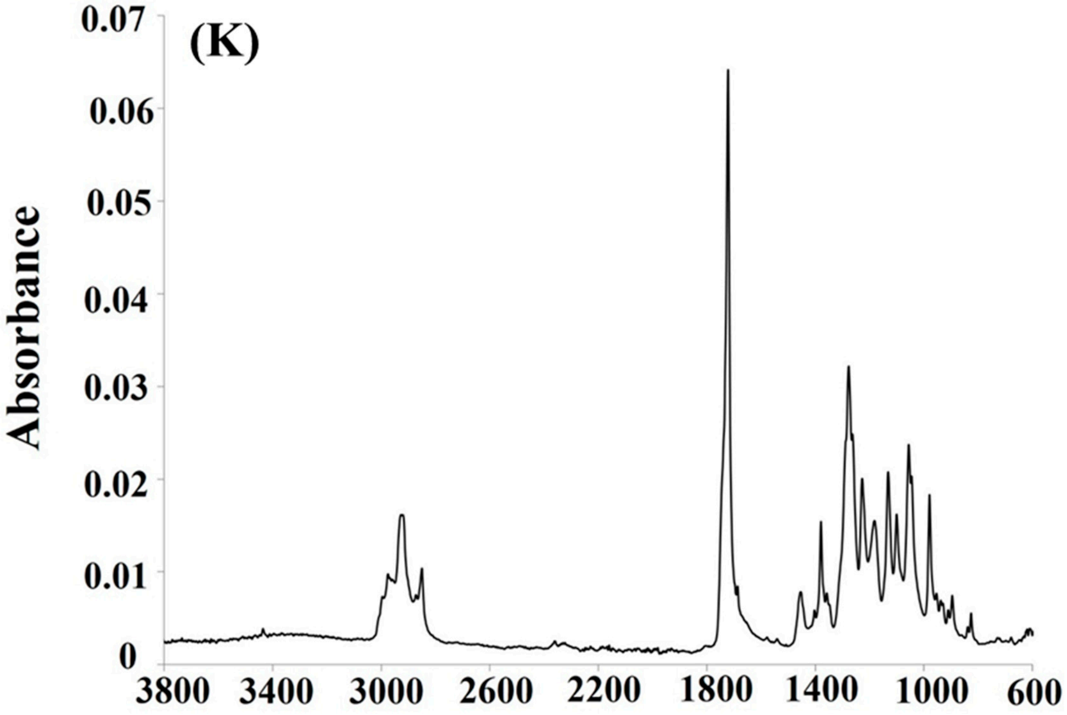
(K)
Absorbance
0.07
0.06
0.05
0.04
0.03
0.02
0.01
0
3800
3400
3000
2600
2200
1800
1400
1000
600
Wavelength (nm)

Figure S2: DSC curves of PHA extracted from *Aeromonas* sp. AC_01 grown in the: non-limited medium supplemented with 1 g/L butyric acid (A), nitrogen-limited medium supplemented with 1 g/L butyric acid (B), non-limited medium supplemented with 2 g/L valeric acid (C), nitrogen-limited medium supplemented with 1 g/L caproic acid (D), non-limited medium supplemented with 0.4 g/L acetic acid : 1.6 g/L butyric acid (E), nitrogen-limited medium supplemented with 0.4 g/L acetic acid : 1.6 g/L butyric acid (F), non-limited medium supplemented with 20% $SMCFAs_{synthetic}$-rich stream (G), nitrogen-limited medium supplemented with 20% $SMCFAs_{synthetic}$-rich stream (H), nitrogen-limited medium supplemented with 30% $SMCFAs_{synthetic}$-rich stream (I), non-limited medium supplemented with 10% $SMCFAs_{extracted}$-rich stream (J), nitrogen-limited medium supplemented with 10% $SMCFAs_{extracted}$-rich stream (K).

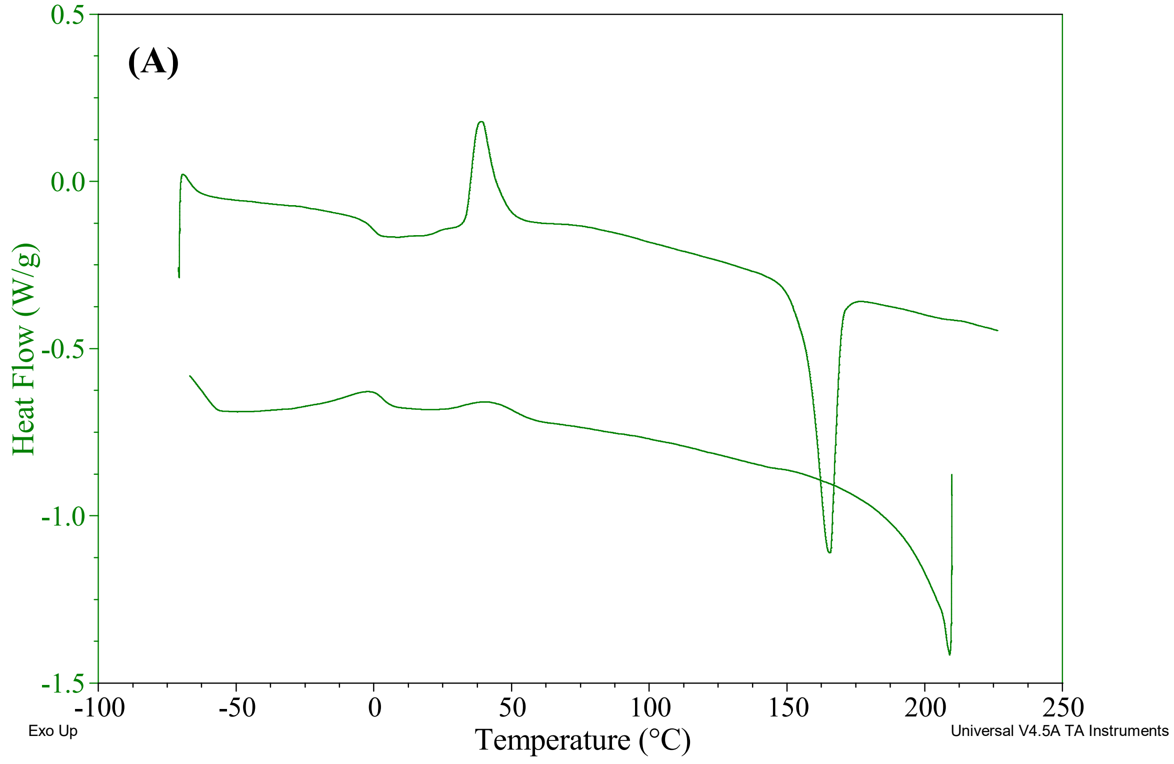

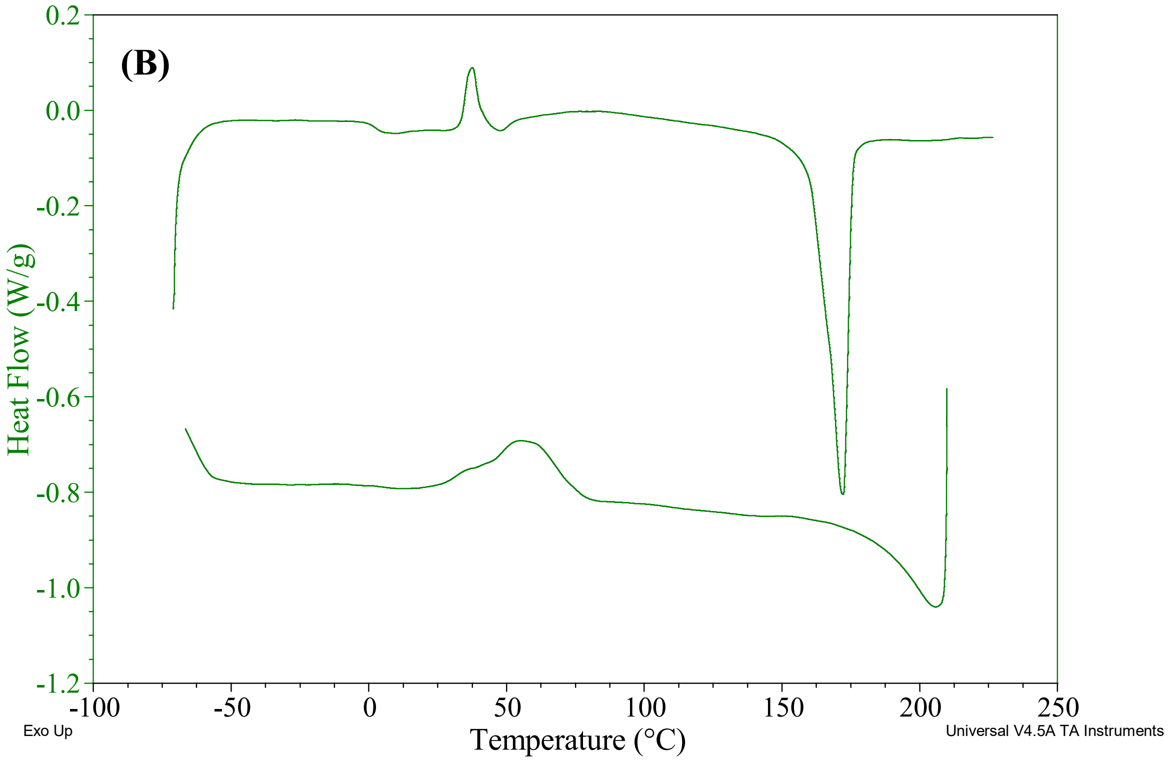
(B)
Heat Flow (W/g)
0.2
0.0
-0.2
-0.4
-0.6
-0.8
-1.0
-1.2
-100
-50
0
50
100
150
200
250
Exo Up
Temperature (°C)
Universal V4.5A TA Instruments

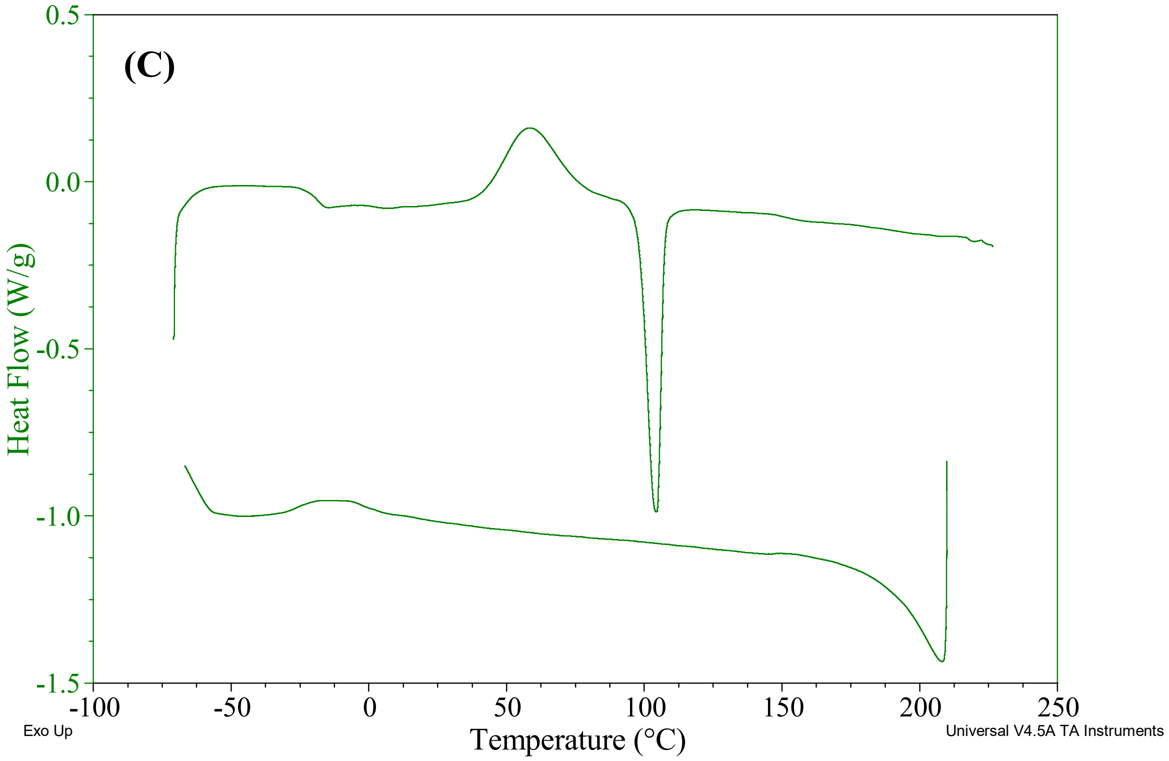
(C)
Heat Flow (W/g)
0.5
0.0
-0.5
-1.0
-1.5
-100
-50
0
50
100
150
200
250
Exo Up
Temperature (°C)
Universal V4.5A TA Instruments

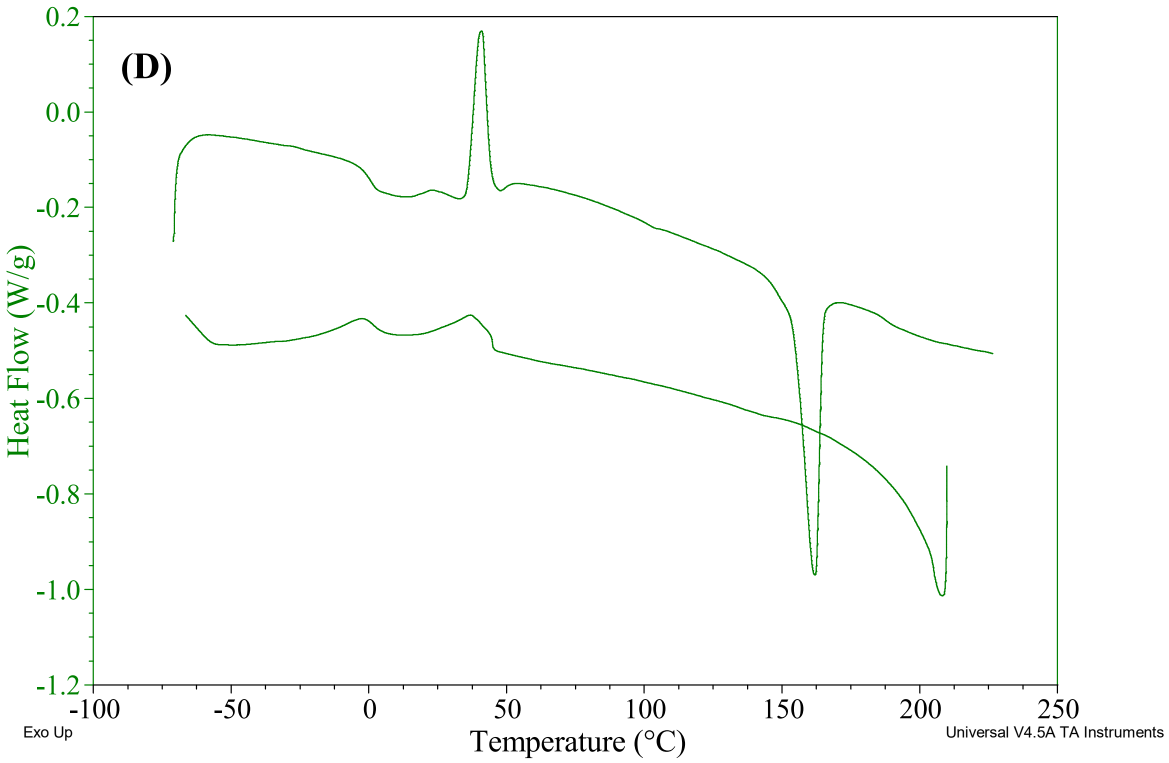
(D)
Heat Flow (W/g)
0.2
0.0
-0.2
-0.4
-0.6
-0.8
-1.0
-1.2
-100
-50
0
50
100
150
200
250
Exo Up
Temperature (°C)
Universal V4.5A TA Instruments

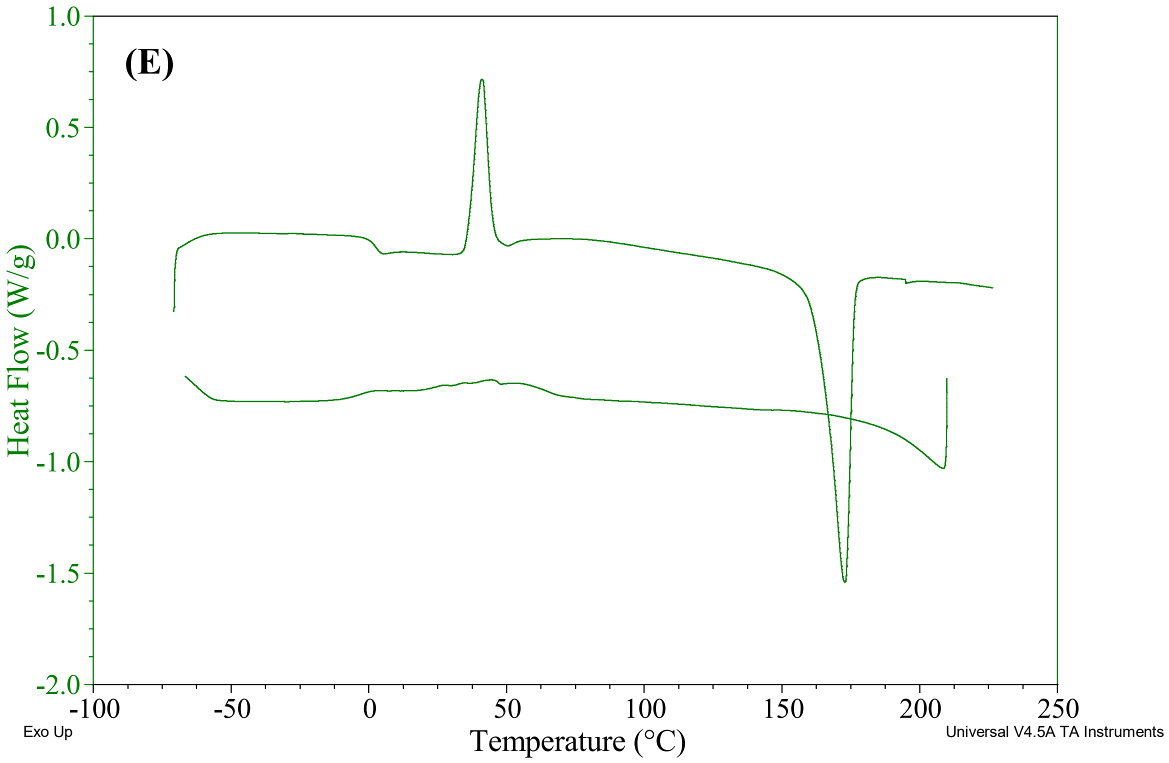
(E)
Heat Flow (W/g)
1.0
0.5
0.0
-0.5
-1.0
-1.5
-2.0
-100
-50
0
50
100
150
200
250
Exo Up
Temperature (°C)
Universal V4.5A TA Instruments

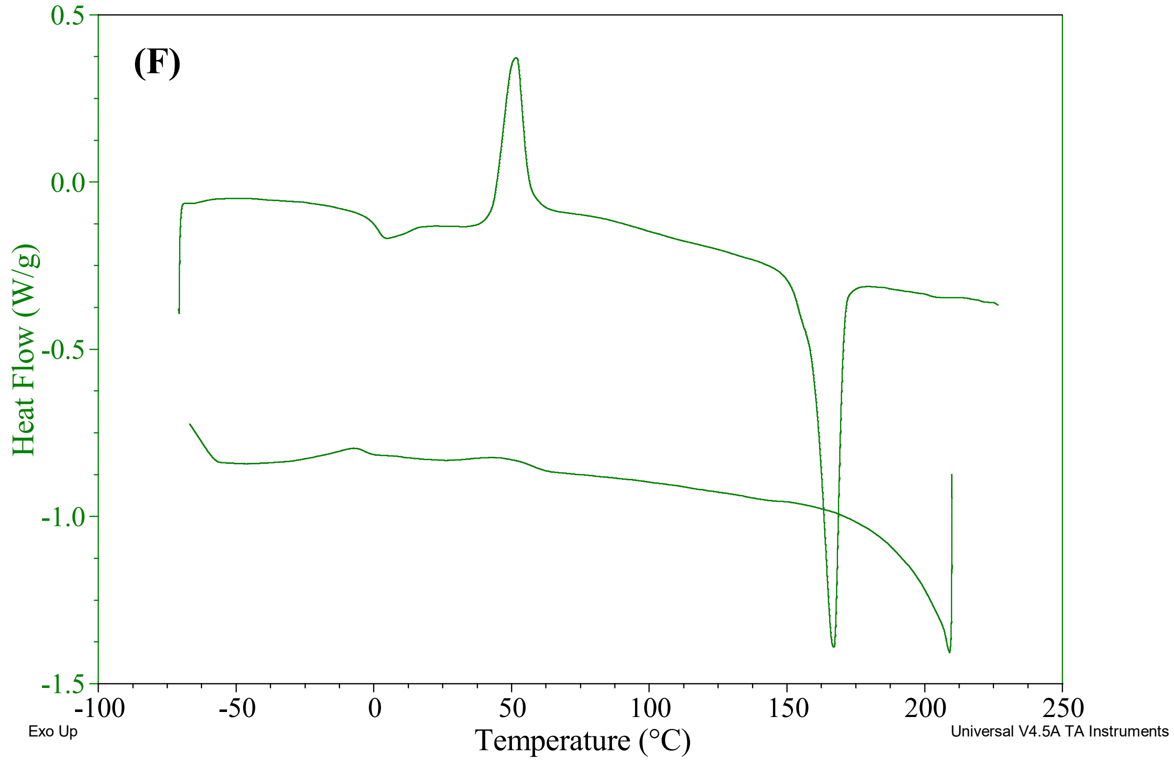

(F)
0.5
0.0
-0.5
-1.0
-1.5
Heat Flow (W/g)
-100
-50
0
50
100
150
200
250
Exo Up
Temperature (°C)
Universal V4.5A TA Instruments

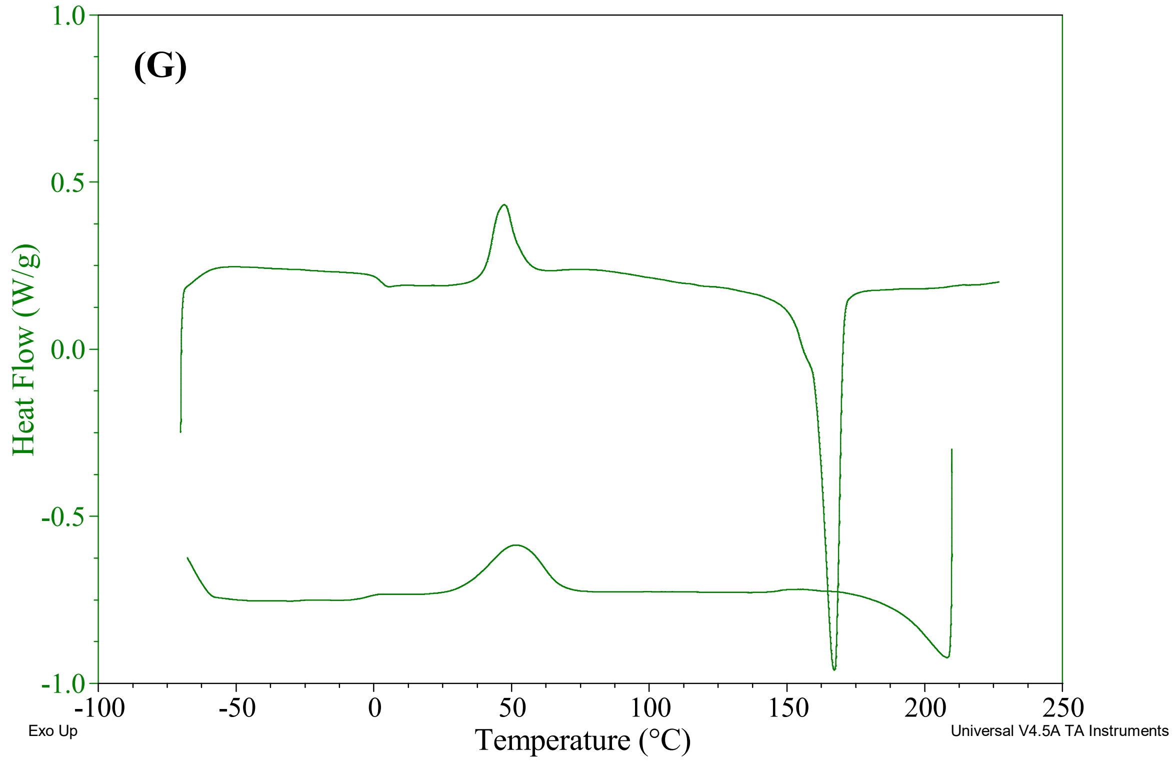

(G)
1.0
0.5
0.0
-0.5
-1.0
Heat Flow (W/g)
-100
-50
0
50
100
150
200
250
Exo Up
Temperature (°C)
Universal V4.5A TA Instruments

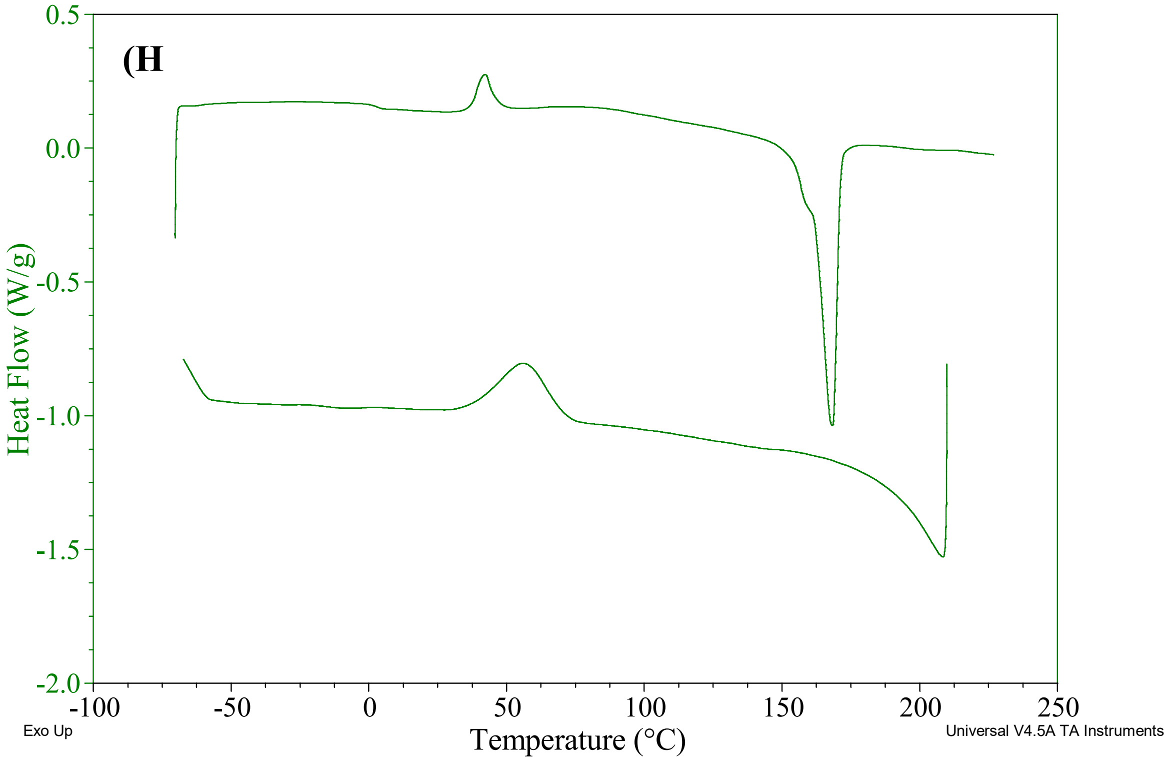
(H
0.5
0.0
-0.5
-1.0
-1.5
-2.0
Heat Flow (W/g)
-100
-50
0
50
100
150
200
250
Exo Up
Temperature (°C)
Universal V4.5A TA Instruments

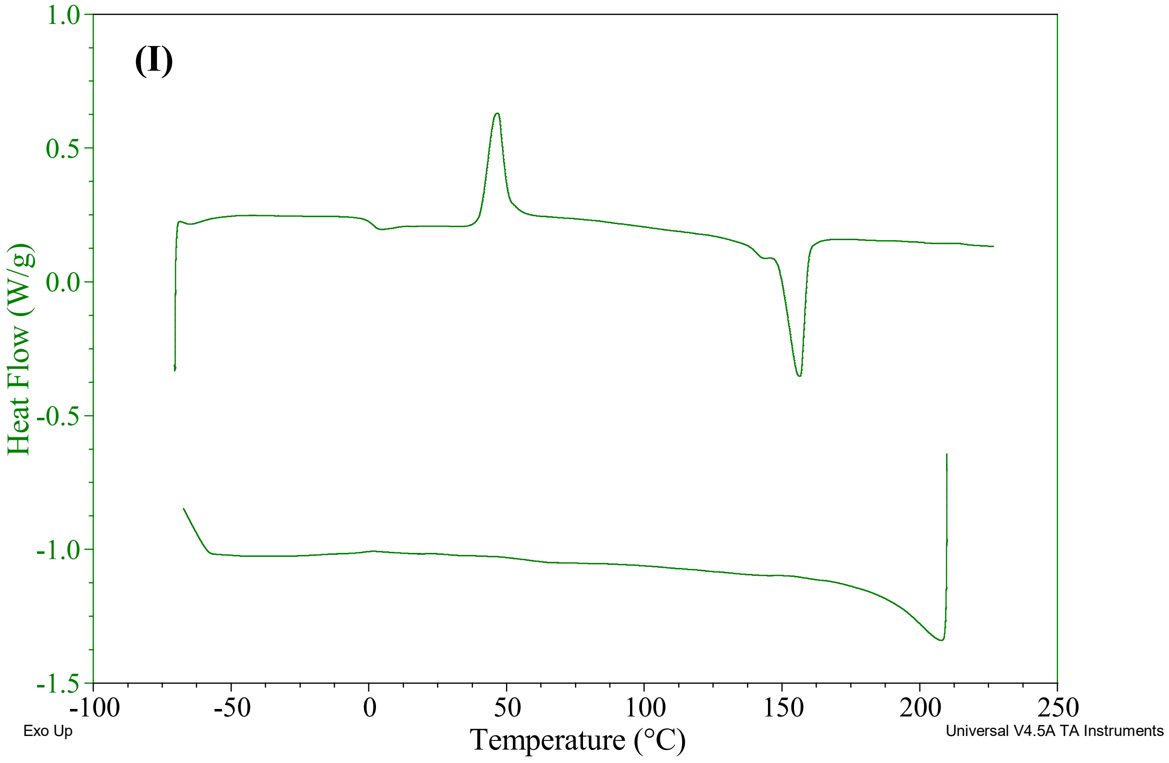
(I)
1.0
0.5
0.0
-0.5
-1.0
-1.5
Heat Flow (W/g)
-100
-50
0
50
100
150
200
250
Exo Up
Temperature (°C)
Universal V4.5A TA Instruments

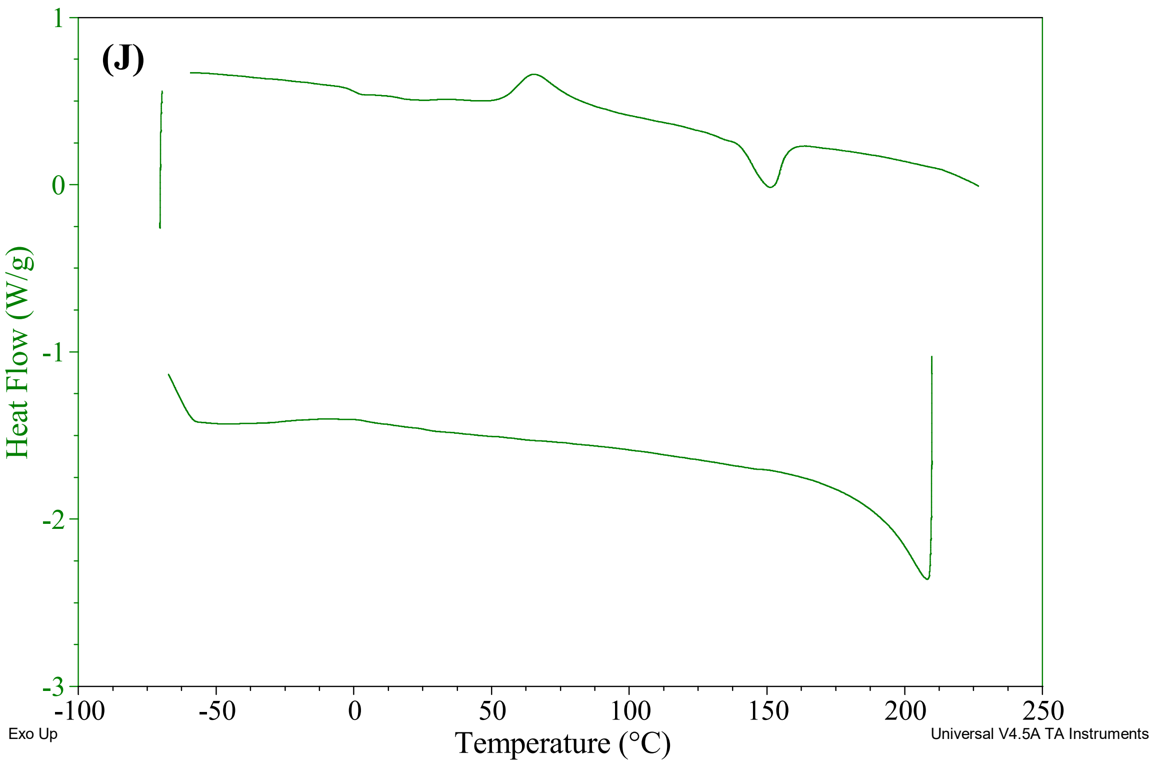
(J)
Heat Flow (W/g)
1
0
-1
-2
-3
-100
-50
0
50
100
150
200
250
Exo Up
Temperature (°C)
Universal V4.5A TA Instruments

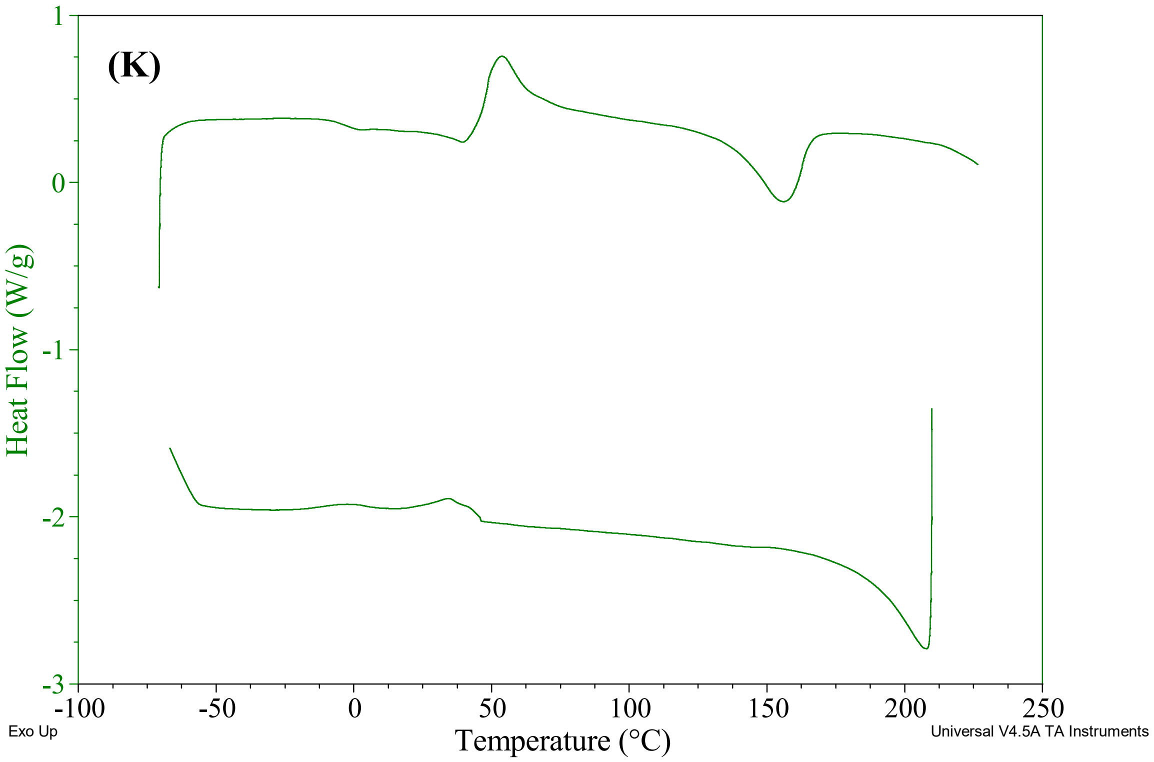
(K)
Heat Flow (W/g)
1
0
-1
-2
-3
-100
-50
0
50
100
150
200
250
Exo Up
Temperature (°C)
Universal V4.5A TA Instruments

Figure S3: TG curves of PHA extracted from *Aeromonas* sp. AC_01 grown in the: : non-limited medium supplemented with 1 g/L butyric acid (A), nitrogen-limited medium supplemented with 1 g/L butyric acid (B), non-limited medium supplemented with 2 g/L valeric acid (C), nitrogen-limited medium supplemented with 1 g/L caproic acid (D), non-limited medium supplemented with 0.4 g/L acetic acid : 1.6 g/L butyric acid (E), nitrogen-limited medium supplemented with 0.4 g/L acetic acid : 1.6 g/L butyric acid (F), non-limited medium supplemented with 20% $SMCFAs_{synthetic}$-rich stream (G), nitrogen-limited medium supplemented with 20% $SMCFAs_{synthetic}$-rich stream (H), nitrogen-limited medium supplemented with 30% $SMCFAs_{synthetic}$-rich stream (I), non-limited medium supplemented with 10% $SMCFAs_{extracted}$-rich stream (J), nitrogen-limited medium supplemented with 10% $SMCFAs_{extracted}$-rich stream (K).

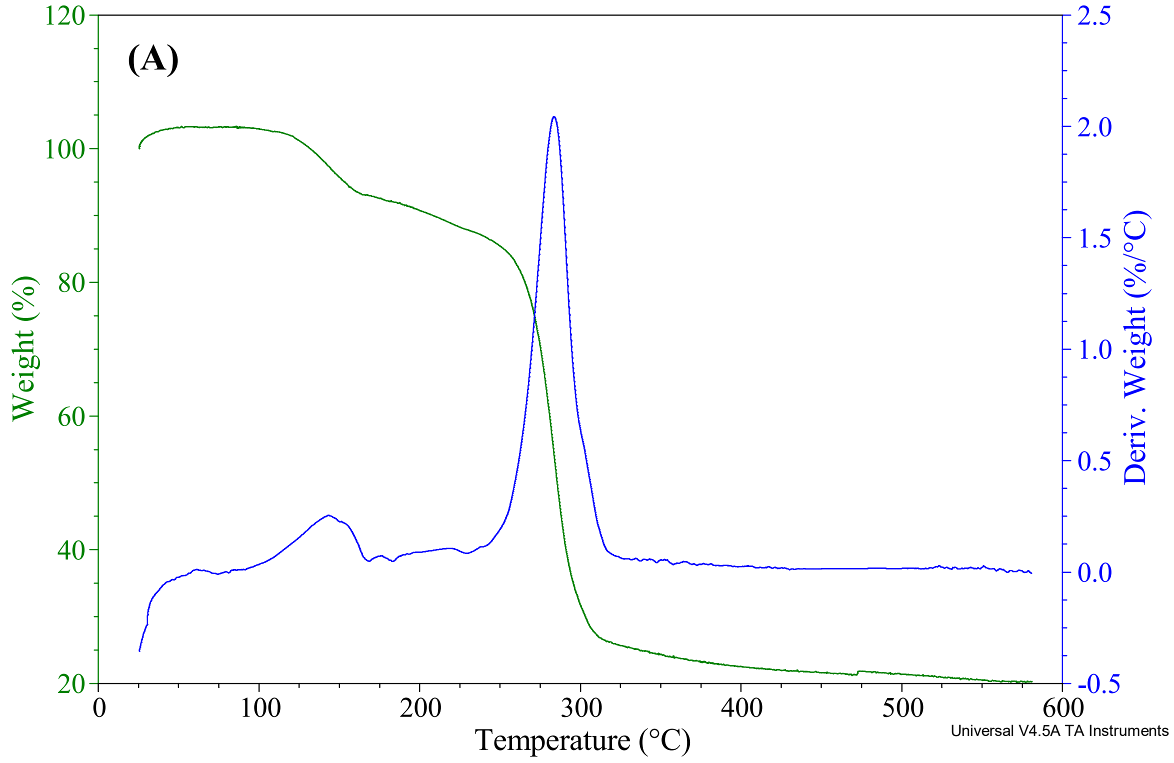

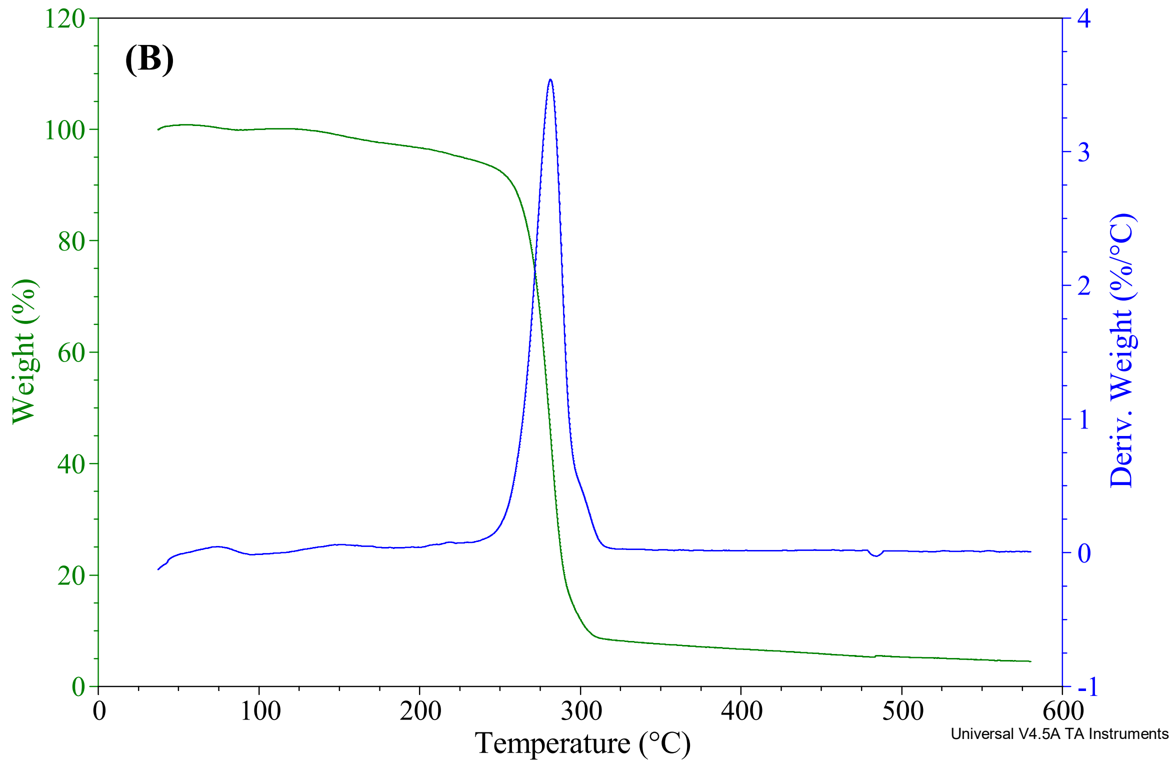
(B)
Weight (%)
Deriv. Weight (%/°C)
Temperature (°C)
Universal V4.5A TA Instruments
0
20
40
60
80
100
120
-1
0
1
2
3
4
0
100
200
300
400
500
600

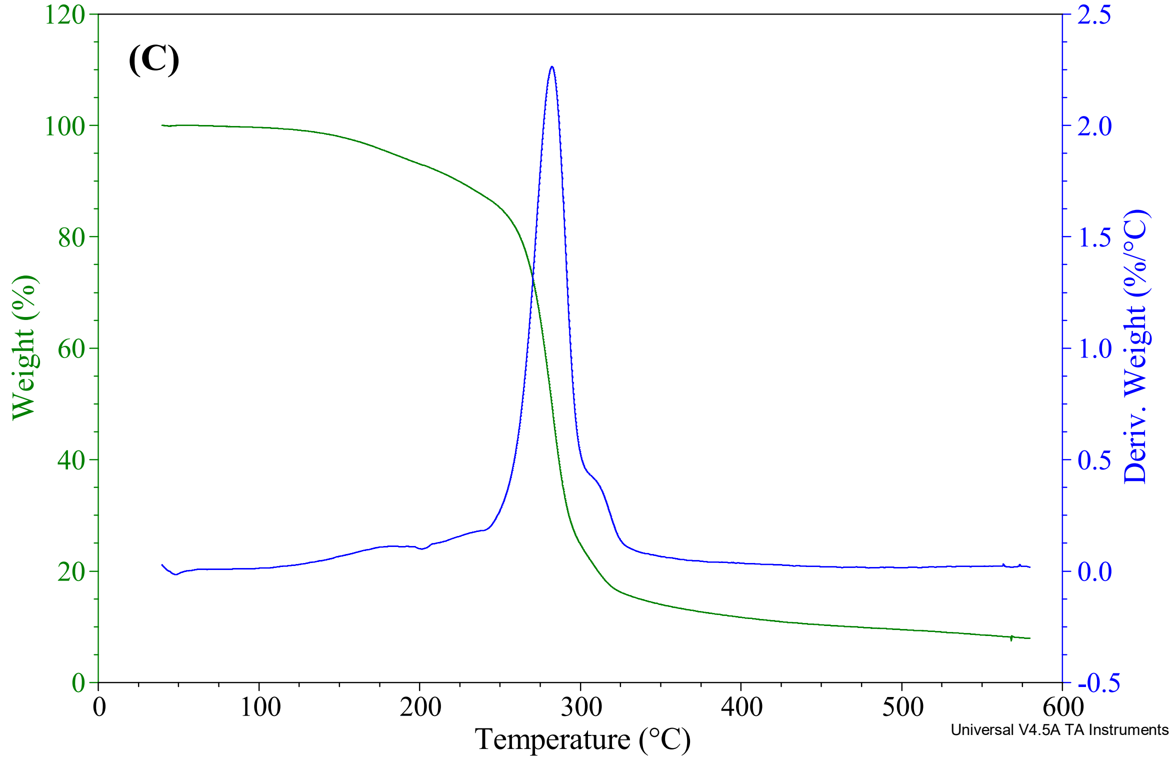
(C)
Weight (%)
Deriv. Weight (%/°C)
Temperature (°C)
Universal V4.5A TA Instruments
0
20
40
60
80
100
120
-0.5
0.0
0.5
1.0
1.5
2.0
2.5
0
100
200
300
400
500
600

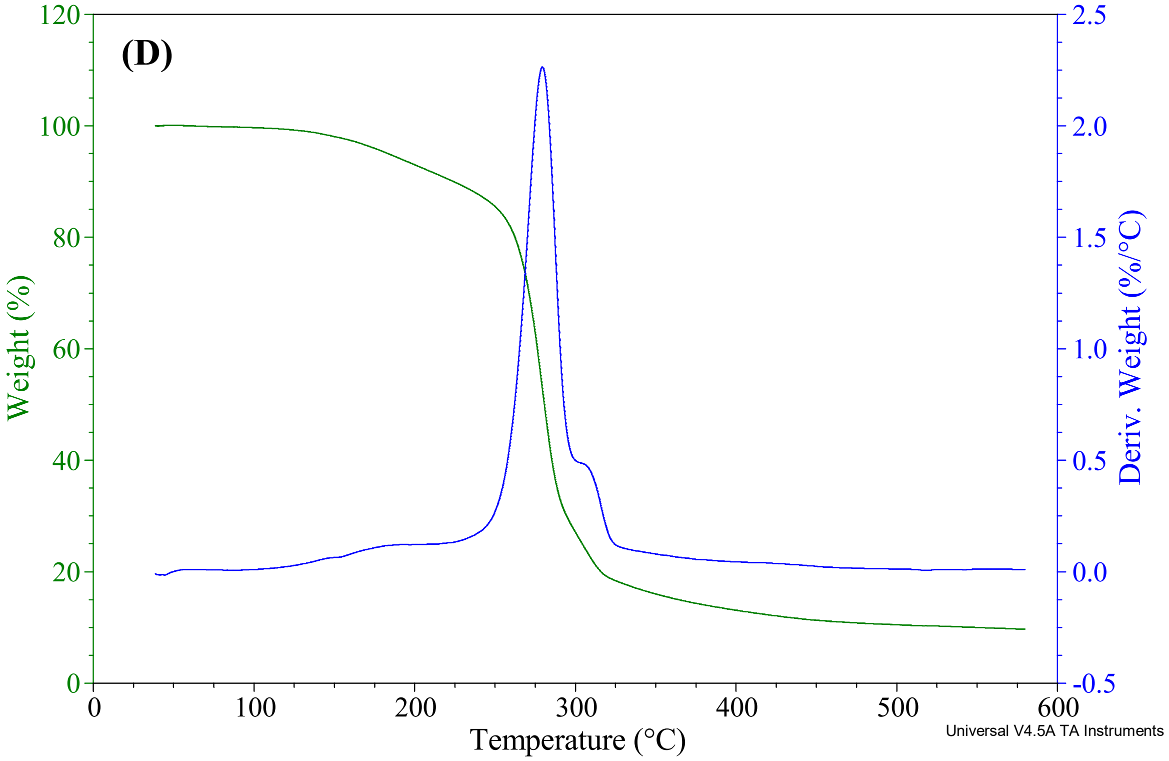

(D)
Weight (%)
Deriv. Weight (%/°C)
Temperature (°C)
Universal V4.5A TA Instruments
0
20
40
60
80
100
120
-0.5
0.0
0.5
1.0
1.5
2.0
2.5
0
100
200
300
400
500
600

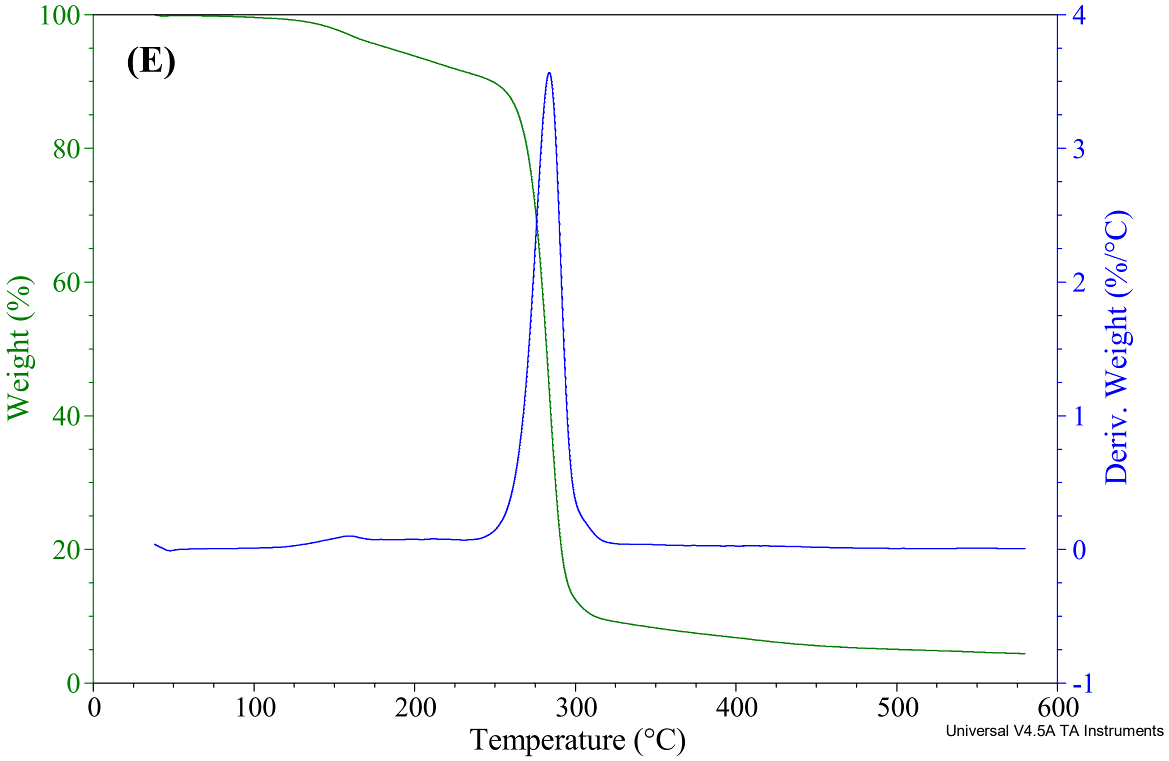

(E)
Weight (%)
Deriv. Weight (%/°C)
Temperature (°C)
Universal V4.5A TA Instruments
0
20
40
60
80
100
-1
0
1
2
3
4
0
100
200
300
400
500
600

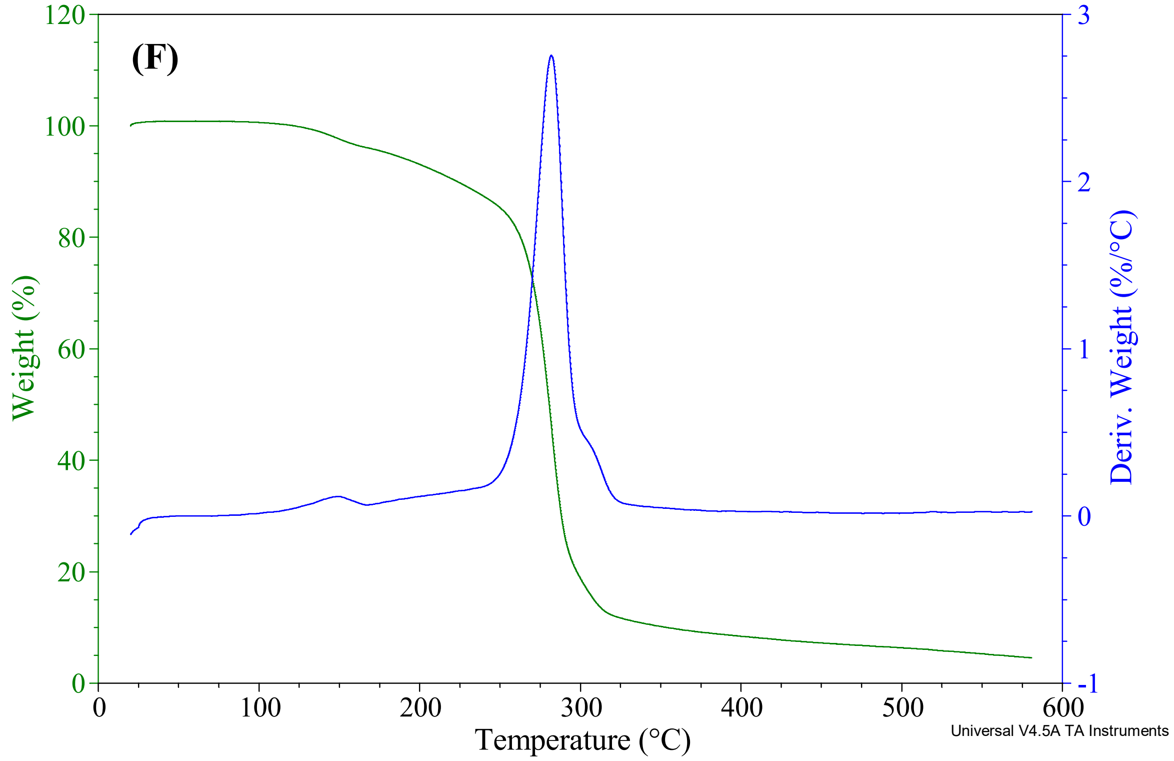

(F)
Weight (%)
Deriv. Weight (%/°C)
Temperature (°C)
Universal V4.5A TA Instruments

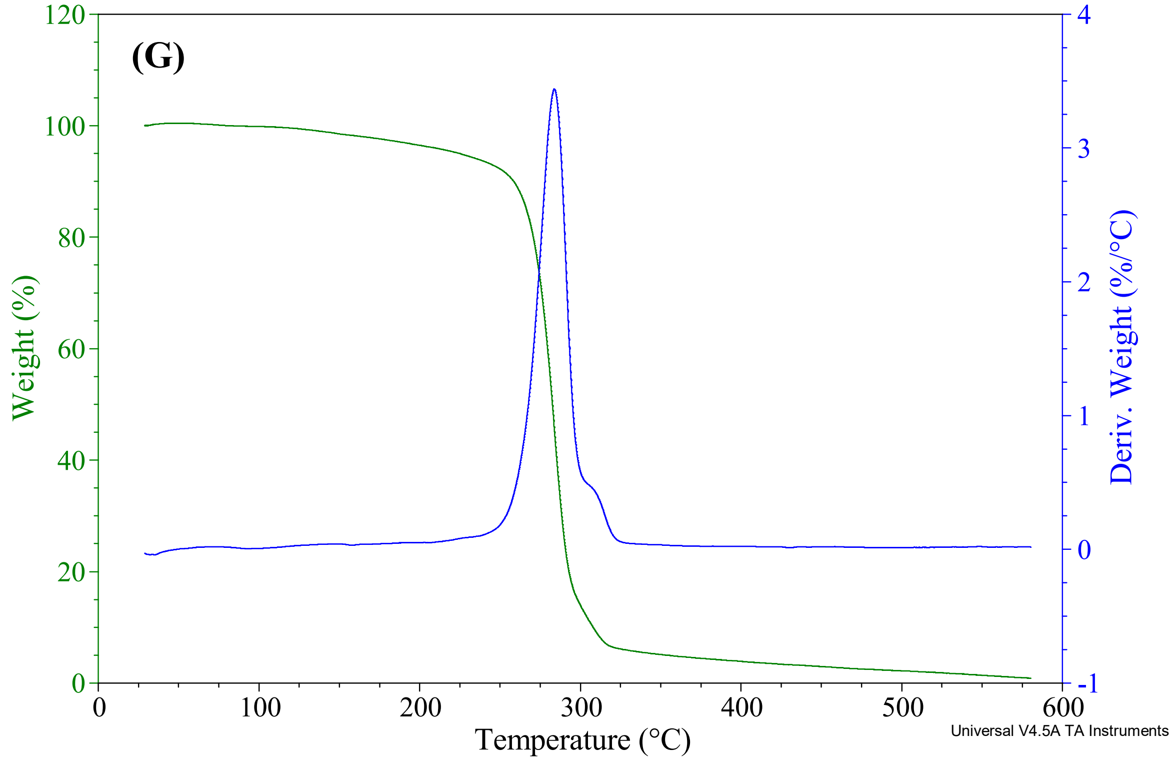

(G)
Weight (%)
Deriv. Weight (%/°C)
Temperature (°C)
Universal V4.5A TA Instruments

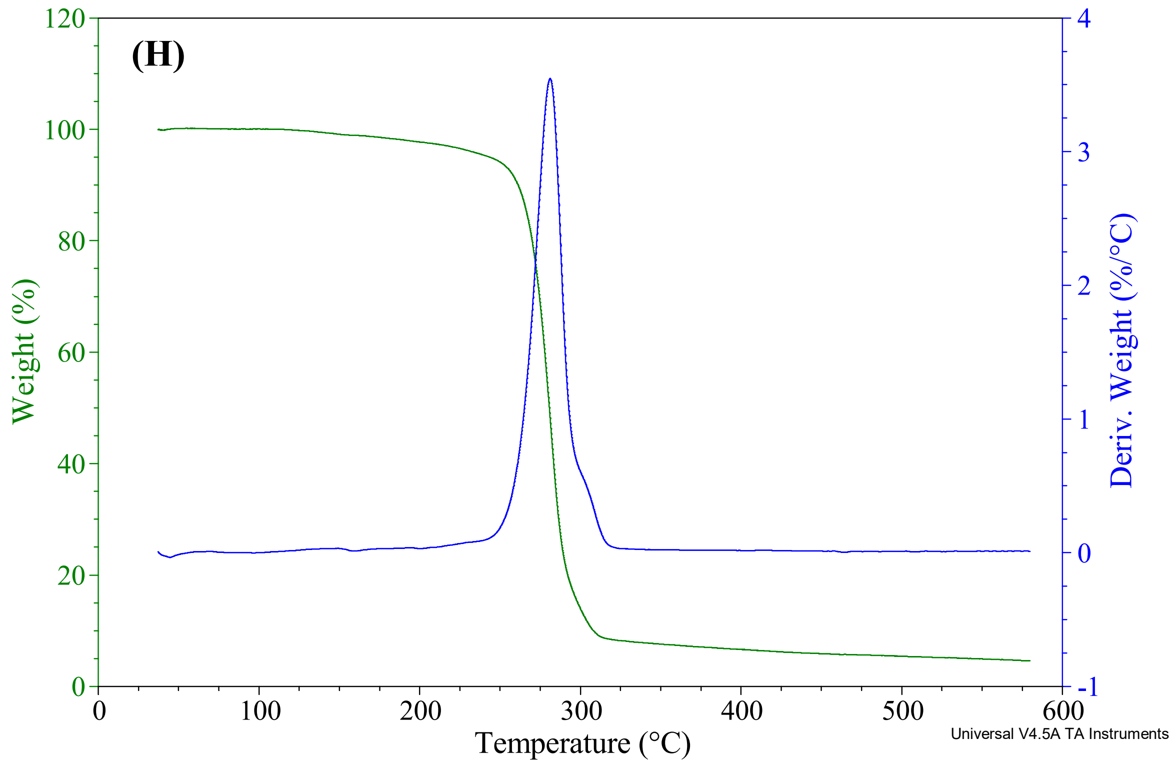
(H)
Weight (%)
Deriv. Weight (%/°C)
Temperature (°C)
0
20
40
60
80
100
120
-1
0
1
2
3
4
0
100
200
300
400
500
600
Universal V4.5A TA Instruments

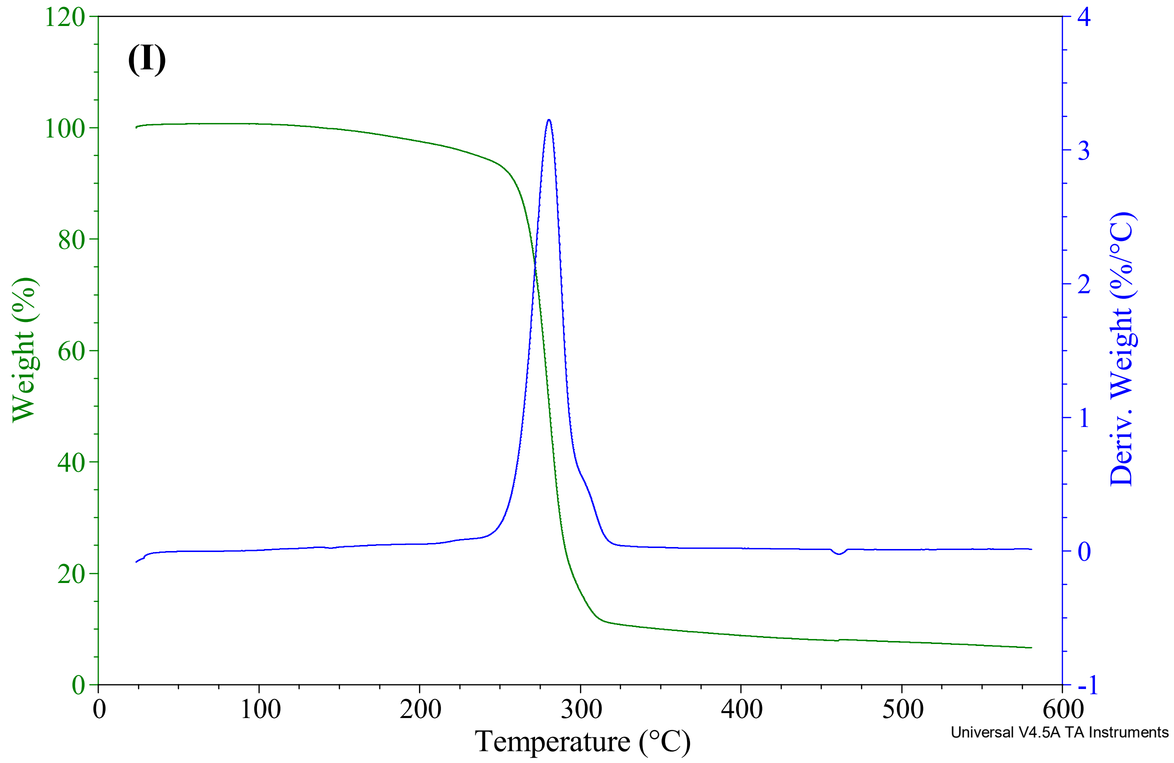
(I)
Weight (%)
Deriv. Weight (%/°C)
Temperature (°C)
0
20
40
60
80
100
120
-1
0
1
2
3
4
0
100
200
300
400
500
600
Universal V4.5A TA Instruments

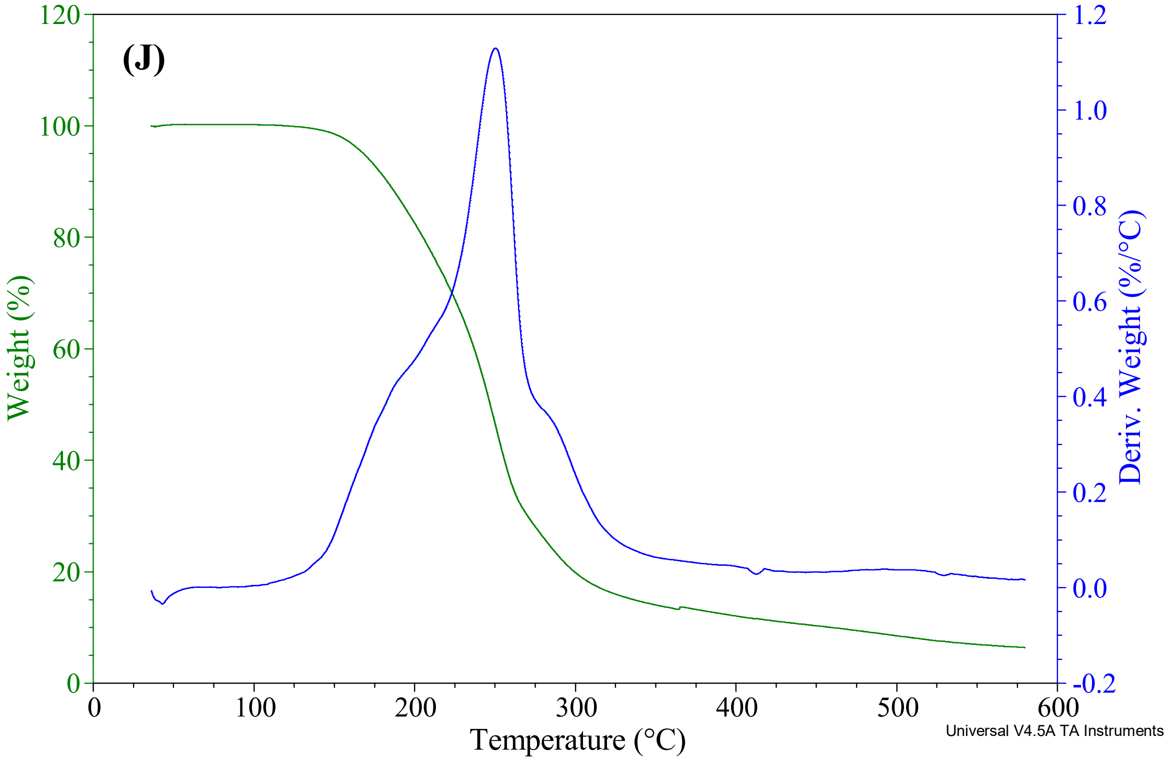
(J)
Weight (%)
Deriv. Weight (%/°C)
Temperature (°C)
0
20
40
60
80
100
120
-0.2
0.0
0.2
0.4
0.6
0.8
1.0
1.2
0
100
200
300
400
500
600
Universal V4.5A TA Instruments

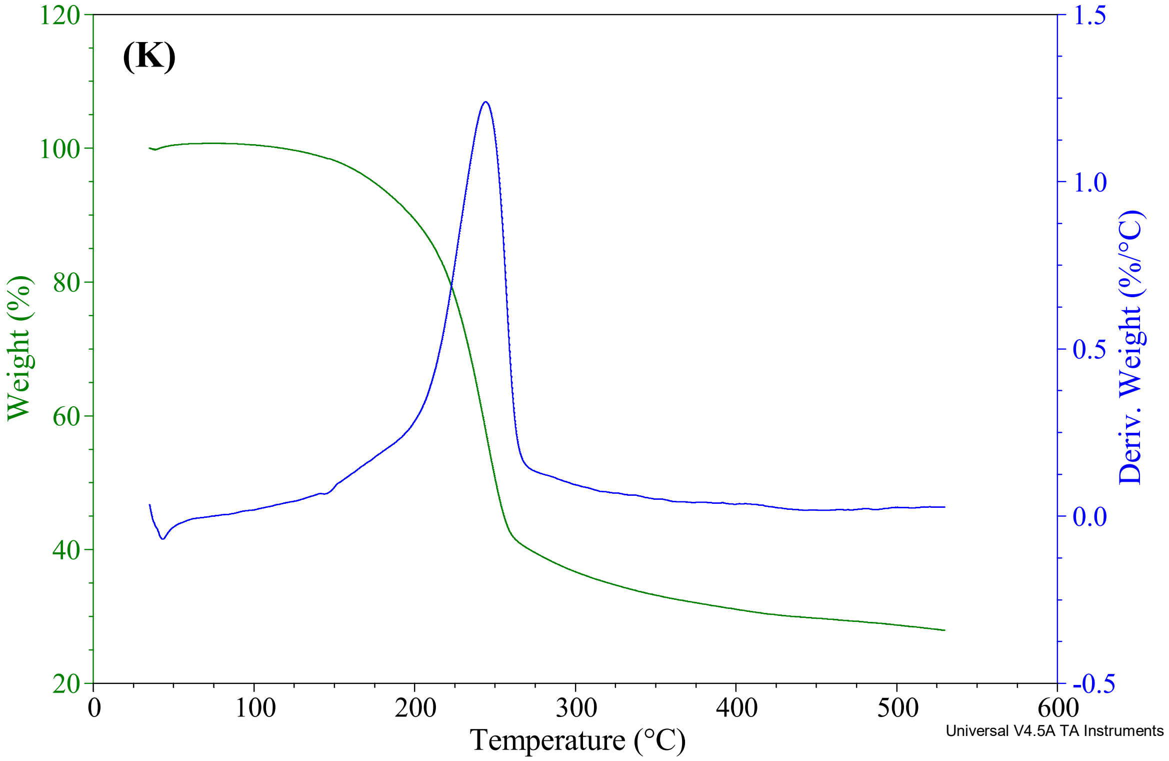
(K)
Weight (%)
Deriv. Weight (%/°C)
Temperature (°C)
20
40
60
80
100
120
-0.5
0.0
0.5
1.0
1.5
0
100
200
300
400
500
600
Universal V4.5A TA Instruments
